# Involvement of abnormal dystroglycan expression and matriglycan levels in cancer pathogenesis

**DOI:** 10.1186/s12935-022-02812-7

**Published:** 2022-12-09

**Authors:** Cristina Quereda, Àngels Pastor, José Martín-Nieto

**Affiliations:** 1 Departamento de Fisiología, Genética y Microbiología, Facultad de Ciencias, Universidad de Alicante, Campus Universitario San Vicente, P.O. Box 99, 03080 Alicante, Spain; 2 Instituto Multidisciplinar para el Estudio del Medio ‘Ramón Margalef’, Universidad de Alicante, 03080 Alicante, Spain

**Keywords:** Dystroglycan, Cancer, Tumorigenesis, Glycosylation, Gene expression, Signal transduction

## Abstract

Dystroglycan (DG) is a glycoprotein composed of two subunits that remain non-covalently bound at the plasma membrane: α-DG, which is extracellular and heavily *O*-mannosyl glycosylated, and β-DG, an integral transmembrane polypeptide. α-DG is involved in the maintenance of tissue integrity and function in the adult, providing an *O*-glycosylation-dependent link for cells to their extracellular matrix. β-DG in turn contacts the cytoskeleton via dystrophin and participates in a variety of pathways transmitting extracellular signals to the nucleus. Increasing evidence exists of a pivotal role of DG in the modulation of normal cellular proliferation. In this context, deficiencies in DG glycosylation levels, in particular those affecting the so-called matriglycan structure, have been found in an ample variety of human tumors and cancer-derived cell lines. This occurs together with an underexpression of the *DAG1* mRNA and/or its α-DG (core) polypeptide product or, more frequently, with a downregulation of β-DG protein levels. These changes are in general accompanied in tumor cells by a low expression of genes involved in the last steps of the α-DG *O*-mannosyl glycosylation pathway, namely *POMT1*/*2*, *POMGNT2*, *CRPPA, B4GAT1* and *LARGE1/2*. On the other hand, a series of other genes acting earlier in this pathway are overexpressed in tumor cells, namely *DOLK*, *DPM1/2/3*, *POMGNT1*, *B3GALNT2*, *POMK* and *FKTN*, hence exerting instead a pro-oncogenic role. Finally, downregulation of β-DG, altered β-DG processing and/or impaired β-DG nuclear levels are increasingly found in human tumors and cell lines. It follows that DG itself, particular genes/proteins involved in its glycosylation and/or their interactors in the cell could be useful as biomarkers of certain types of human cancer, and/or as molecular targets of new therapies addressing these neoplasms.

## Dystroglycan and dystroglycanopathies

**Fig. 1:**
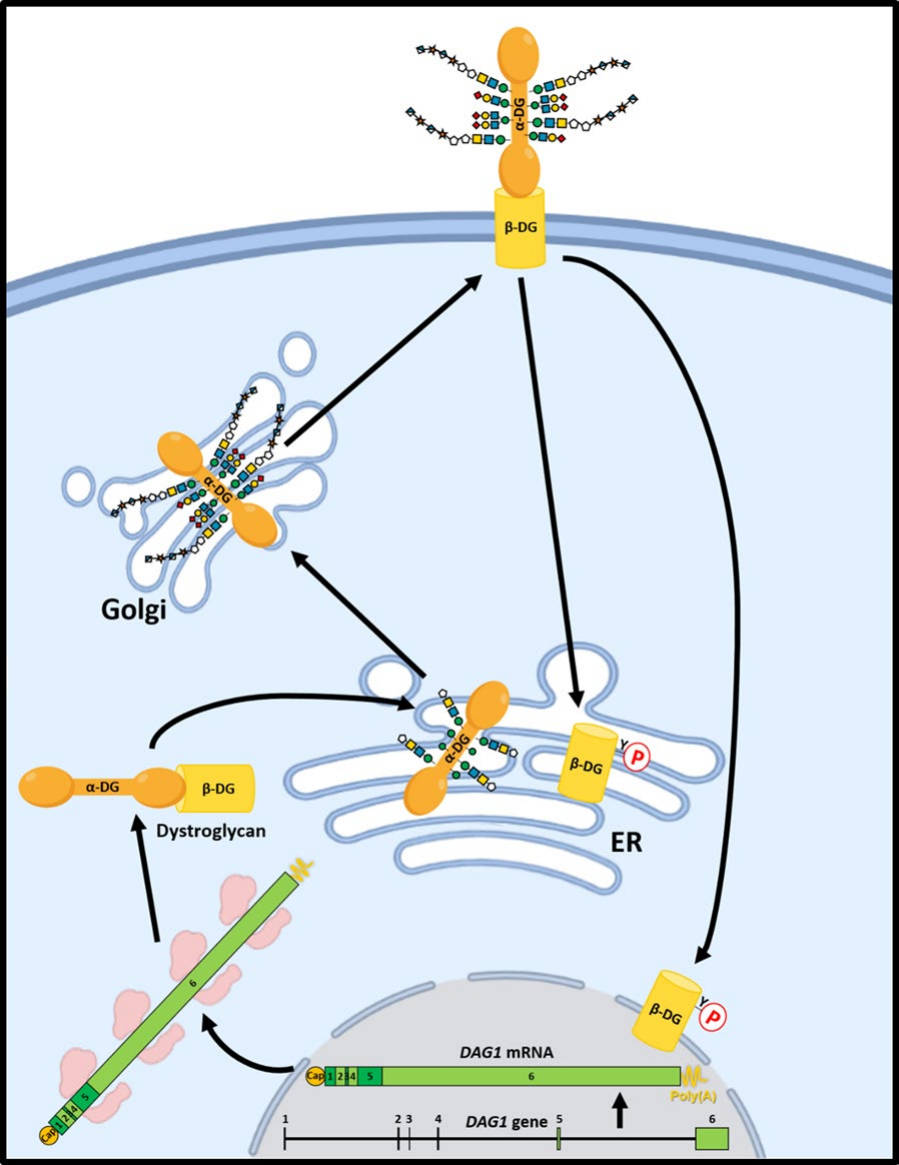
Dystroglycan gene structure, biosynthesis and intracellular transport. DG is encoded by the *DAG1* gene, which is composed of six exons and five introns, and is transcribed into a single mRNA. The encoded polypeptide undergoes post-translational cleavage in the endoplasmic reticulum (ER) in an autocatalytic fashion to yield two subunits, α-DG and β-DG. Then *O*-mannosyl glycans are added to the mucin-like region of α-DG in the ER and Golgi complex, and the two subunits are targeted to the plasma membrane, where they remain non-covalently bound. Upon cell-ECM interaction the β-DG subunit becomes phosphorylated, allowing its dissociation from α-DG and its retrograde targeting to the ER and the nucleus, where it modulates nuclear structure and activity, including the expression of genes involved in normal cell proliferation. Created with BioRender.com

Dystroglycan (DG) was discovered in the embryonic chicken brain as the predominant plasma-membrane glycoprotein able to bind laminin [1], the major component of the extracellular matrix (ECM) basal lamina [2]. DG is encoded by the *DAG1* gene, which is transcribed into a single 5.7 kb mRNA whose protein product exhibits 895 amino acids (aas) and a molecular mass of 97.5 kDa in our species. This polypeptide undergoes post-translational cleavage in an autocatalytic fashion at residue Ser-654 to yield two subunits, α-DG and β-DG, which remain non-covalently associated [3–6] (Fig. 1). The primary structure of DG is highly conserved in vertebrates and expressed in a wide variety of fetal and adult tissues, including among others muscle, neural, adipose, epithelial, endothelial and blood, albeit being more prevalent in the skeletal muscle and brain [3, 7–10]. Once removed its signal peptide, the mature α-DG subunit (aas 30–653; ∼74 kDa) is a membrane-associated extracellular polypeptide formed by two globular N- and C-terminal domains separated by a mucin-like region rich in *O*-linked sugar chains, among them *O*-mannosyl glycans, and also containing three *N*-linked glycosylation sites [3, 11–13]. As a result of extensive *O-*glycosylation of its core polypeptide, α-DG is detected in western blots as a broad band ranging in size between 100 and 190 kDa, depending on the tissue under study [3, 14–16]. As a difference, the β-DG subunit (aas 654–895; ∼43 kDa) is an integral transmembrane polypeptide containing a single *N-*glycosylated residue and lacking *O-*glycosylation sites [3, 6]. The non-covalent association of α-DG and β-DG occurs within the so-called SEA domain, located extracellularly between the DG mucin-like (ECM-binding) and transmembrane domains [5].

DG is an integral component of the so-called dystrophin-glycoprotein complex (DGC), which links ECM proteins such as laminin and proteoglycans to the actin cytoskeleton via dystrophin in skeletal muscle cells and non-muscular tissues [17–19]. This complex also contains sarcoglycans (in the muscle), dystrobrevins, syntrophins, sarcospan and dystrophin, and includes other peripheral members or associated proteins, such as neuronal-type nitric oxide synthase (nNOS) and caveolin 3 [10, 11, 20]. The DGC is thought to contribute to the structural stability of the muscle cell membrane during cycles of contraction and relaxation, thereby protecting the muscle from stress-induced membrane damage [15, 21]. In the DGC, α-DG is responsible for the cell’s attachment to the ECM by doubly interacting non-covalently with β-DG by virtue of its C-terminal domain, and in a Ca^2+^-dependent fashion with ECM components, such as laminin, agrin, perlecan and biglycan in muscle and neural tissues, neurexin and Slit in the brain, and pikachurin in the retina, by virtue of *O-*mannosyl glycans linked to its mucin-like domain [16, 22–25]. The cytosolic portion of β-DG, i.e., its C-terminal domain, is anchored to the actin cytoskeleton through its interaction with dystrophin or utrophin, the latter a dystrophin paralog found in non-muscular tissues and at the neuromuscular junction [26, 27]. This domain includes the so-called PPxY^892^ motif at its very C-terminus, which interacts with the WW domain of dystrophin/utrophin and the SH3 domain of Grb2. Upon phosphorylation of the Tyr-892 residue, β-DG becomes unable to interact with WW and SH3 domains, thereby losing its linkage to the cytoskeleton [28].

To date, 22 genes have been identified which encode, in addition to DG itself (*DAG1* gene), a set of known or putative glycosyltransferases and other enzymes, mostly residing in the endoplasmic reticulum or the Golgi complex (Fig. 1), and which are directly or indirectly involved in the addition of *O-*mannosyl glycans to the mucin-like region of α-DG by sequentially acting along a branched, complex glycosylation pathway [5, 25, 26, 29–32]. Loss-of-function mutations in these genes, such as *POMT1*, *POMT2*, *POMGNT1*, *POMGNT2*, *FKTN* (encoding fukutin), *FKRP*, *LARGE1* and many others [19, 25, 29, 32–35], of which *SLC35A1* [36], *INPP5K* [37], *TRAPPC1* and *GOSR2* [38] have been the latest identified, result in impairments in the glycosylation of α-DG, which diminishes or abolishes α-DG binding affinity to its extracellular ligands. The complex *O*-mannosyl glycan structure harbored by α-DG is composed of three core glycans, named M1, M2 and M3, of which the ECM-binding motif, designated as matriglycan, is added to the phosphorylated core M3 glycan [35, 39]. Such motif consists of a polysaccharide composed of the repeating disaccharide unit (Xylα1-3GlcAβ1-3)_n_ that is synthesized by the enzyme products of genes *B4GAT1* and *LARGE1* [40]. In humans, a deficient glycosylation of α-DG underlies a spectrum of recessive genetic disorders referred to as dystroglycanopathies (DGPs). These are congenital muscular dystrophies with associated central nervous system (CNS) abnormalities characterized by progressive muscular dystrophy frequently coursing with brain malformation, intellectual disability and a panoply of ocular defects [41–44]. From higher to lower severity, DGPs include the Walker-Warburg syndrome, muscle-eye-brain disease (MEB), Fukuyama congenital muscular dystrophy, moderate congenital muscular dystrophies and limb-girdle muscular dystrophies [27, 41–43].

In addition to muscle and neural cells, DG is expressed in many other cell types, all of which share the property of being in direct contact with basement membranes in their residing tissue [9, 45]. Many different studies have illustrated that α-DG plays a role in epithelialization, myogenesis and neurogenesis during embryonic development and postnatally, including the assembly of basement membranes, cell shape and polarity formation, tissue integrity maintenance, and cell survival and differentiation in many tissues [8, 14, 17, 23, 25, 28, 30, 46–48]. Also, DG serves as a receptor for several members of the arenavirus family, by virtue of high-affinity binding to α-DG of their envelope glycoproteins, and for the prokaryote *Mycobacterium leprae* indirectly through its ability to bind laminin, allowing the entrance of these pathogens into human cells [17, 39, 49, 50]. Furthermore, as a DGC component β-DG serves as a scaffold for the organization of adhesion structures, integrin-mediated signal transduction systems, including the phosphatidylinositol 3-kinase (PI3K)/AKT and extracellular signal-regulated kinase (ERK)/mitogen-activated protein kinase (MAPK) (also known as Raf/Ras/MEK/ERK) pathways [27, 51–53], and modulation of nuclear structure and function (Fig. 1) [54, 55], as explained in this review.

## Cancer hallmarks and dystroglycan

It is estimated that over 19 million new cancer cases and nearly 10 million cancer deaths occurred in 2020 worldwide according to the Global Cancer Observatory (http://gco.iarc.fr). The most frequently diagnosed tumor types from higher to lower incidence are breast, lung, colorectum, prostate, stomach, liver, esophagus and cervix. Altogether, they represented ca. 57% of the global incidence cancer cases in 2020 and together with pancreas cancer they constitute the top nine most deadly cancers in the world today, being lung cancer the most frequent cause of cancer death in males and breast cancer in females [56].

As normal cells evolve progressively to a neoplastic state, they acquire distinctive and complementary capabilities that enable tumor growth and metastatic dissemination. There are two main features enabling cells to develop cancer phenotypic abilities: genomic instability (generating random mutations including chromosomal rearrangements) and inflammatory state (driven by cells of the immune system), which in turn promote tumor progression by diverse mechanisms [57]. The most fundamental trait of cancer cells is their ability to sustain chronic proliferation, which they achieve by acquiring the self-ability of constitutive division-promoting signaling by producing growth-factor ligands themselves or by stimulating the delivery of growth factors from normal cells within the supporting tumor-associated stroma [57–59].

Another hallmark capability of cancer cells is their ability to evade the actions of tumor-suppressor genes. Most prominent among the regulators disrupted in cancer cells are the retinoblastoma/RB protein and p53, two prototypical transcription factors playing a pivotal role in regulatory circuits determining the cells’ decisions to proliferate or, alternatively, undergo senescence and apoptosis [57, 60]. Since programmed cell death serves as a natural barrier to cancer development, tumor cells have evolved a variety of strategies to limit or circumvent apoptosis [57]. In addition, they acquire an unlimited replicative potential in order to generate macroscopic tumors, being telomerase activity in this light correlated with a resistance to the induction of senescence and apoptosis [57, 61, 62]. Alike healthy tissues, tumors need nutrients and O_2_ for their sustenance, together with an ability to clear metabolic wastes and CO_2_. Such needs are fulfilled by the tumor-associated neovasculature, which is generated by a process of angiogenesis [57].

Regarding mechanisms underlying invasiveness, carcinomas arising from epithelial tissues can progress to achieve higher pathological grades of malignancy, reflected in local invasion and distant metastasis. Cancer cells may alongside develop alterations in their shape and in their ability to attach to other cells and the ECM [57]. The expression of genes encoding cell-cell and cell-ECM adhesion molecules turns altered in this process, typically becoming downregulated in a process resembling the so-called epithelial-mesenchymal transition (EMT) physiologically occurring during embryonic development [63]. The multistep process of invasion and metastasis is therefore a succession of changes in cell biology, starting with local invasion and followed by intravasation of cancer cells into proximal vessels, their transit through the hematogenous and lymphatic systems, their extravasation into distant tissues with formation of micrometastases, and finally the growth of these into macroscopic tumors, or colonization [57, 64].

During cancer progression, primary tumor cells escape from their normal growth control by modifying the binding affinities of their cell membrane receptors. In a wide sense, unregulated proliferation and invasion are often accompanied by dramatic alterations in the expression pattern of molecules active at the interface between the ECM basement membrane and the cell’s plasma membrane [65, 66]. In this context, DG has been reported to act as a crucial receptor required for the development and maintenance of epithelial, muscular and neural tissues, among many others [9, 14, 28, 46]. Here, laminin binding to α-DG regulates cell shape via reorganization of the actin cytoskeleton, modulates tissue-specific gene expression and promotes cell survival and differentiation over proliferation and migration. These functions are all affected during tumor progression, in a growing list of human neoplasias and tumor-derived cell lines [14, 67–69], and in this context given the pivotal role of α-DG in the formation of stable contacts by cells with ECM molecules, its loss of function may constitute an effective mechanism by which cancer cells’ adhesion to the ECM becomes weakened or even lost [65, 66, 69]. Deficiencies in α-DG expression and, more frequently, glycosylation have been detected by using antibodies recognizing as epitopes *O*-mannosylated carbohydrate structures on α-DG forming part of matriglycan (such as IIH6 and VIA4-1) for both western blotting and immunohisto/cytochemistry analyses. These alterations have been reported in a wide variety of tumor types and cell lines, and have been associated with tumorigenesis contributed by loss of the cells’ link to the ECM [27, 52, 70, 71]. Additionally, the β subunit of DG is a versatile protein considered as a receptor for cell-ECM interaction and acting as a multifunctional platform for cytoskeletal remodeling, cell-adhesion dynamics, signal transduction and modulation of nuclear morphology [52, 55]. In this context, it has been shown that upon α-DG–laminin interaction, β-DG becomes phosphorylated on its residue Tyr-892 by the (non-receptor) protein tyrosine kinase c-Src [72, 73], this allowing β-DG binding to SH2 domain-containing proteins, such as the growth factor receptor-bound adaptor protein Grb2 [27, 52]. Additionally, this promotes β-DG dissociation from the DGC and its import from the cytoplasm into the nucleus, where it exerts a modulatory role of nuclear architecture and activity, including gene expression, in response to extracellular cues [52, 54, 74]. Alterations in these regulatory processes are being detected in a growing variety of human tumor cells, especially in epithelial-derived cancers.

In this work we review current literature on the relationship between DG and cancer, which has noticeably declined over the last decade. This review intends to compile all the existing information on alterations detected in the expression and function of DG and the genes/proteins involved in its glycosylation in human tumors and tumor-derived established cancer cell lines. In addition to the involvement of deficiencies in α- and/or β-DG expression, and α-DG glycosylation (i.e., matriglycan levels), in the pathogenesis of cancer, alterations in the expression levels of particular DGP-associated genes and proteins are discussed in the light of the specific tumor and cell-line types in which they have arisen. Finally, possible implications of these findings in the unraveling of new molecular biomarkers and potential therapeutical targets of cancer are emphasized in a biomedical context.

## Deficiencies in dystroglycan expression and glycosylation in human tumors

A significant number of human primary tumors of many different types have been found to exhibit low or undetectable DG expression, in terms of *DAG1* mRNA, α-DG and/or βDG protein levels, and/or low or undetectable glycosylated α-DG levels, which are compiled in Table 1. Such tumors are here dealt with according to their human-body system or apparatus of origin.

**Table 1:** Alterations in dystroglycan expression and glycosylation in human tumors

Alteration	Levels	Tumor type	References
*DAG1* mRNA	Underexpressed	Oral squamous cells	[75]
		Kidney	[76]
		Acute myeloid leukemia (AML)	[77]
α-DG (core) protein	Underexpressed	Prostate	[78]
		AML	[77]
α-DG glycosylation	Hypoglycosylated/unglycosylated	Oral squamous cells	[75, 79–81]
		Esophagus	[82]
		Stomach	[83]
		Pancreas	[84]
		Colon	[85, 86]
		Prostate	[87–89]
		Cervix	[90]
		Vulva	[90]
		Kidney	[76, 91–93]
		Breast	[85]
		Glioma (III/IV)	[66, 94, 95]
		Medulloblastoma	[93]
		Retinoblastoma	[93]
		Neuroblastoma	[93]
		AML	[77]
		Osteosarcoma	[93]
		Rhabdomyosarcoma	[93, 96]
β-DG protein	Underexpressed	Oral squamous cells	[79]
		Esophagus	[82, 97]
		Stomach	[83]
		Pancreas	[84]
		Colon	[85, 97]
		Prostate	[78, 87, 98, 99]
		Kidney	[76]
		Ureter	[97]
		Breast	[85, 97, 98]
		Glioma (III/IV)	[94, 95]
		Medulloblastoma	[93]
		Retinoblastoma	[93]
		AML	[77]
		Osteosarcoma	[93]

The table compiles all alterations reported so far in human tumors in the expression of dystroglycan at the mRNA (*DAG1* gene) or protein (α- or β-DG subunit) level, or in the glycosylation status of α-DG. Tumor types and references reporting these results are also indicated

### Digestive system

In squamous cell carcinomas of the oral mucosa, the expression of the *DAG1* gene has been analyzed at the mRNA level, and has been found undetectable compared to its levels in normal oral squamous epithelium [75]. Also, undetectable or very low levels of glycosylated α-DG have been reported in other studies on oral cell carcinomas [75, 79–81]. Regarding β-DG, it showed an anomalous staining pattern in this cancer type, with most tumors completely lacking immunolabeling and a minority exhibiting an increased signal depending on the tumor and area analyzed. Additionally, β-DG revealed the appearance of species (or ‘proteoforms’) with different molecular masses on western blots, of 43 kDa (normal size) and 30–31 kDa (∼31 kDa hereafter), together with an 80 kDa band from improperly-processed DG protein [79].

In addition to oral carcinomas, alterations in DG have been found in other tumors of the digestive system. In esophageal cancer, no significant differences were observed in *DAG1* mRNA levels between normal and tumor tissue. However, a complete loss of α-DG glycosylation (i.e., matriglycan) has been found in nearly all samples studied, with a large reduction in glycosylated α-DG levels in the rest of them [82]. Also, an absent or reduced expression of β-DG has been detected in all the esophageal adenocarcinomas analyzed [82, 97].

Undetectable α-DG matriglycan levels have been reported in primary gastric cancer, together with reduced expression of the β-DG subunit [83]. Furthermore, this loss of α-DG glycosylation was correlated with tumor progression and patient survival. Thus, tumors with lower levels of α-DG matriglycan compared to adjacent healthy tissues were prognostic of poor patient survival, compared to tumors with levels of glycosylated α-DG similar to those in the surrounding tissue [83]. A similar correlation could not be established, however, between a lower patient survival and underexpression of β-DG.

As in other tumors of the digestive system, almost undetectable glycosylated α-DG and β-DG protein have been found in pancreatic ductal carcinoma, together with a replacement of β-DG membrane localization by cytoplasmic staining [84]. It must be noted, however, that such α-DG hypoglycosylation and β-DG underexpression were not directly related to downregulation of *DAG1* mRNA, that was concluded not to be statistically significant, which suggested that altered post-transcriptional modifications, such as glycosylation, might result in defective protein processing or sorting [84]. It was also found that patients with tumors displaying a lower expression of glycosylated α-DG exhibited a lower mean post-operative survival than patients with higher levels. However, an analogous inverse correlation between survival and β-DG expression could not be established, as it was also the case for gastric cancer samples [83, 84].

Other studies have unveiled that in colon cancer α-DG glycosylation is nearly absent and β-DG is underexpressed compared to healthy tissue [85, 86, 97]. Also, similarly to pancreatic cancer [84], the mean overall survival of patients with colon tumors exhibiting decreased levels of α-DG matriglycan was lower than that of patients with higher glycosylation levels [86]. Regarding β-DG, its expression has been found reduced or undetected in nearly all colon cancer samples examined [97]. It was also reported that the decrease of both glycosylation of α-DG and expression of β-DG (as assessed by western blotting) correlated with tumor grade and Duke’s stage together with the appearance of the aforementioned ∼31 kDa β-DG band additional to the full-length 43 kDa band [85], which has been determined to arise from proteolytic cleavage of the β-DG extracellular domain [27]. Therefore, loss of DG is an event with prognostic significance in colon cancer.

### Genitourinary system

Among tumors of the reproductive system, prostate cancer constitutes one of the most studied regarding DG. By using an antibody to the core protein of α-DG, it was concluded that this polypeptide was underexpressed (by −5.26-fold) in samples from elderly prostate cancer patients, and the same accounted for β-DG [78]. In other studies, the expression of β-DG was found also decreased (by −6.67-fold) in prostate adenocarcinoma samples [78, 87, 98, 99]. Regarding α-DG, its glycosylation was nearly absent in prostate cancer, with patient samples exhibiting an increased Gleason score (reflecting the tumor grade of growth or spread) detectably showing lower α-DG matriglycan levels [87–89]. The decrease in the levels of glycosylation and expression of DG was associated with the aggressiveness of the tumor together with a loss of integrity of the ECM [87]. Even, the observed reduction of α-DG glycosylation was greater than loss of β-DG expression, both in terms of a low immunoreactivity, which suggested that the first was not a consequence of α-DG (core) downregulation [87]. Furthermore, in these patients β-DG proteoforms harboring sizes of 43, ∼31 and 26 kDa were detected by western blotting in the cytoplasmic and nuclear fractions of both normal and prostate tumor tissue samples [99]. Interestingly, it was observed that the ∼31 kDa fragment, containing the β-DG transmembrane and cytoplasmic domains [100, 101] and phosphorylated on tyrosine (residue Tyr-892), was more frequently translocated to the nucleus [99].

In other types of tumors of the reproductive system, such as cervical and vulvar cancers, hypoglycosylation of α-DG has also been detected (by −3.23- and −1.82-fold, respectively). A progressive, significant decrease of the percentage of cells exhibiting glycosylated α-DG has been observed in correlation with the degree of tumor progression in squamous intraepithelial lesions and invasive cervical carcinomas, as compared to the fraction of cells positive for glycosylated α-DG quantitated in normal cervical epithelium [90]. The same was true for cells with glycosylated α-DG scored in vulvar intraepithelial neoplasia and invasive vulvar carcinoma in comparison to normal vulvar epithelium. In contrast, β-DG exhibited normal expression levels, with both 43 and ∼31 kDa species being detectable [90].

In studies of tumors of the urinary tract, the *DAG1* mRNA was found to be downregulated (by −2.17-fold) in clear cell renal cell carcinoma [76]. Furthemore, hypoglycosylation of α-DG was reported in samples of renal cell carcinomas [76, 91, 92], although differences in the expression of β-DG were not so evident or relevant [92]. Likewise, in a study on pediatric solid tumors, it was determined that in different samples of Wilms tumor (a malignant neoplasia of the kidney) α-DG was hypoglycosylated while β-DG presented normal expression levels [93]. Conversely, expression of the β-DG subunit was detected decreased or absent in ureteric transitional cell carcinomas [97].

### Breast cancer

In breast cancer, a progressive reduction of α-DG matriglycan and β-DG expression has been reported in correlation with increasing tumor grade and stage [85]. Also, it was determined that tumor stage and (low) patient survival was directly correlated with a loss of matriglycan on α-DG, although not with tumor grade, invasion or recurrence. Conversely, an inverse correlation was detected between the levels of glycosylated α-DG and the expression of the proliferation marker Ki-67 and tumor suppressor protein p53 [85]. Other studies have as well reported a reduced or absent expression of β-DG in samples of mammary tumors [97, 98].

### CNS tumors

Regarding tumors of the nervous system, diminished immunoreactivity from glycosylated α-DG (by ‒2.62-fold) and from β-DG protein (by ‒0.60-fold) has been reported in malignant, grade III and IV gliomas, the latter also designated as glioblastomas [66, 94, 95]. Additionally, it was determined that levels of matriglycan were inversely correlated with tumor grade, recurrence and cancer-related death being more frequent in patients whose tumors exhibited low levels of glycosylated α-DG [95], although this issue is somewhat controversial [102]. Glycosylation of α-DG almost undetectable and underexpression of β-DG have also been evidenced in medulloblastoma and retinoblastoma samples from patients, while in western blots of neuroblastoma samples α-DG glycosylation was not detected and β-DG species of different sizes higher than 43 kDa were found, which are likely to represent uncleaved αβ-DG polypeptides [93].

### Other tumor types

In tumors of the hematopoietic system, DG alterations have been studied in patients with acute myeloid leukemia (AML). Here, the *DAG1* mRNA was found to be downregulated (by −2.17-fold) in leukemia primary blasts with different degrees of maturation (subtypes M1, M2 and M3), as compared to control (CD34^+^) stem-progenitor cells from healthy individuals [77]. An underexpression of α-DG core protein (by −3.57-fold) was also detected in AML blast cells, together with α-DG hypoglycosylated levels (by −4.17-fold) on western blots. However, the underexpression of β-DG (by −1.85-fold) and its change of location seem to be more relevant in this type of cancer, this protein translocating from the cytoplasm to the nucleus upon becoming phosphorylated on Tyr-892 in leukemia cells, as a difference with blasts from healthy individuals where it remained restricted to the plasma membrane and cytoplasm [77].

In tumors of the musculoskeletal system, a reduction in glycosylated α-DG levels has only been found in Ewing’s sarcoma (a rare cancer developing in bones or surrounding soft tissue) and rhabdomyosarcoma (a malignant tumor of striated muscle), although in the latter α-DG (core) protein levels were not found altered, and β-DG was found expressed at different sizes, of 43 kDa and higher [93, 96]. By contrast, a hypoglycosylation of α-DG was detected in osteosarcomas along with underexpression of β-DG [93].

## Deficiencies in dystroglycan expression and glycosylation in human cancer cell lines

Given the wide variety of tumors in which the levels of expression and/or glycosylation of DG have been found altered, investigations have been carried out in a large number of model cell lines representative of such variety of tumors, in order to better understand the role of DG in cancer progression. Table 2 enlists all the human established cell lines studied so far in which alterations in the DG (α and/or β subunit) expression and/or matriglycan levels have been reported.

**Table 2:** Alterations in dystroglycan expression and glycosylation in human cancer cell lines

Alteration	Levels	Tumor type	Cell line	References
*DAG1* mRNA	Underexpressed	Acute promyelocytic leukemia (APL)	HL-60	[77, 85]
		Acute myeloid leukemia (AML)	Kasumi-1	[77]
α-DG (core) protein	Underexpressed	Glioblastoma	U87MG	[66]
			A172MG	
		APL	HL-60	[77, 85]
		AML	Kasumi-1	[77]
α-DG glycosylation	Hypoglycosylated/unglycosylated	Oral squamous cells	SCC-4	[79, 81]
			SCC9-, -15, -25	[79]
			CAL27	[81]
		Esophagus	FLO-1	[82]
		Pancreas	PANC-1	[87]
			BxPC-3	[87, 103]
		Colon	HT29	[100]
			LoVo	[104]
		Prostate	PC3	[87, 88, 103, 105]
			PC3-L	[105]
			TEM4-18	[87, 103]
			LNCaP	[87, 88, 103, 105]
			DU145	[85, 100, 105]
		Cervix	HeLa	[69]
		Kidney	A498	[103]
		Breast	MCF7	[67, 85, 100, 105, 106]
			T4-2	[67]
			BT549	[85, 106]
			MDA-MB-231, −435, −453, −468	[67, 69, 87, 103, 105–107]
			MDA-MB-134VI, -436	[106]
			CAMA1	
			LY2	
			HCC38, 70, 1143, 1419, 1500, 1937, 1954	
			HS578T	
			AU565	
			SUM52PE, 149PT, 159PT	
			ZR75B	
		Glioblastoma	U87MG	[66, 106]
			U251MG	[106]
			A172MG	[66]
			LN-18	[106, 107]
			LN-229	[106]
		APL	HL-60	[77, 85]
		AML	Kasumi-1	[77]
		Melanoma, skin	BLM	[108]
			M14	
			IF6	
		Rhabdomyosarcoma	PCB82, 232, 380	[96]
			Rh18	
		Lung	H1299	[69]
			H2030	
β-DG protein	Underexpressed	Esophagus	FLO-1	[82]
		Colon	HCT116	[85, 100]
			SW620	
		Prostate	DU145	[85, 88, 100, 109]
		Breast	MCF7	[85, 100]
			T4-2	[67]
			MDA-MB-134VI	[106]
			CAMA1	
			LY2	
			HCC1419, 1500, 1569, 1954	
			BT474	
		APL	HL-60	[77, 85]
		AML	Kasumi-1	[77]
	Overexpressed	Breast	MCF7	[110]
			MDA-MB-453	
		Melanoma, skin	IF6	[108]

The table compiles all alterations reported so far in human cancer cell lines in the expression of dystroglycan at the mRNA (*DAG1* gene) or protein (α- or β-DG subunit) level, or in the glycosylation status of α-DG. The tissue of origin of each cell line and references reporting these results are also indicated

### Digestive system

Regarding tumors of the digestive system, several oral squamous cell carcinoma (SCC)-derived cell lines have been analyzed, with the result that glycosylated α-DG was undetectable [79, 81]. At the molecular level, two β-DG bands were observed on western blots analyses, of 43 and ∼31 kDa, the latter arising from proteolysis carried out (although not uniquely) by matrix metalloproteinases (MMPs) [79].

In other studies, it was determined that the esophageal adenocarcinoma cell line FLO-1 showed a reduction in the levels of α-DG matriglycan and β-DG expression, although the *DAG1* mRNA was found to be normally expressed [82]. Also, α-DG matriglycan was absent in the pancreatic cancer cell lines PANC-1 and BxPC-3 [87, 103]. Finally, in colon cancer studies, glycosylated α-DG was undetectable in the HT29 and LoVo cell lines together with the appearance of the ∼31 kDa β-DG species in these and two other colon cancer cell lines, namely HCT116 and SW620 [85, 100, 104]. The full-length, 43 kDa β-DG was nearly absent in these two cell lines, which again did not correlate with alterations in *DAG1* mRNA expression [85, 100].

### Genitourinary system

Among tumors of the reproductive system, there exists a wide variety of studies on prostate cancer cell lines. In the highly-metastatic prostate cell line PC3, as well as in the less-metastatic cell lines LNCaP and DU145, α-DG matriglycan levels were found to be undetectable [85, 87, 88, 100, 103, 105], despite normal levels of the *DAG1* mRNA, which led to the proposal of a post-transcriptional regulatory mechanism being operative in these cell lines [88]. Noticeably, the two PC3-derived cell lines PC3-L and TEM4-18, which exhibited undetectable levels of glycosylated α-DG, were even more aggressive than their parental, PC3 cell line. However, at variance with what occurred in samples from patients with prostate cancer, α-DG core protein was detected at normal levels in these cell lines [87, 103, 105]. In addition, β-DG expression was nearly absent only in the DU145 cell line [85, 88, 100, 109], although it was found fragmented (with sizes of 43, ∼31 kDa and others) in the PC3, LNCaP and DU145 cell lines [85, 88, 100, 102, 109].

Regarding the cervical cancer HeLa cell line, although the α-DG core protein was detected, its glycosylation levels were found reduced [69]. Yet, β-DG was normally expressed, albeit displaying the ∼31 kDa species [69, 100], as described in the samples of patients with cervical cancer [90]. Lastly, regarding tumor cell lines of the urinary system, studies on α-DG alterations have only been made in the kidney cell line A498, where α-DG was found to be hypoglycosylated [103].

### Breast cancer

To date, breast cancer is the tumor type most studied regarding DG alterations in cell lines. Glycosylated α-DG was found to be undetectable or nearly absent together with virtually undetected β-DG levels in MCF7 [67, 85, 100, 105, 106], T4-2 [67], MDA-MB-134VI and six additional breast cancer cell lines [106]. As well, glycosylation of α-DG alone (i.e., without analyzing β-DG expression) was absent in five cell lines of the MDA-MB series [67, 69, 85, 87, 103, 105–107], as well as in BT549 and ten additional breast cancer cell lines [85, 106], all of them compiled in Table 2. In the MCF7 and BT549 lines such alterations have been analyzed together with *DAG1* mRNA expression, with normal levels of the latter being detected, again suggesting regulation at the post-transcriptional level [85]. Regarding β-DG, cell lines have been found that, while displaying α-DG hypoglycosylation, exhibited either normal or reduced expression of β-DG, as stated above, although with the latter showing the ∼31 kDa species in addition to the full-length 43 kDa band [67, 85, 100]. On the other hand, a slight β-DG upregulation and cytoplasmic accumulation of especially the ∼31 kDa proteoform have been detected in the MCF7 and MDA-MB-453 cell lines, likely due to post-transcriptional regulation carried out by the protein disulfide isomerase AGR2 [110].

### CNS tumors

Undetectable expression levels of α-DG core protein in parallel to α-DG hypoglycosylation have been found in cell lines derived from human glioblastomas, namely U87MG and A172MG [66, 106]. Also, glycosylated α-DG was nearly absent in the glioblastoma cell lines U251MG, LN-18 and LN-229 [106, 107]. However, as a difference with tumor samples obtained from patients, all glioblastoma cell lines studied exhibited the ∼31 kDa β-DG species in addition to the normal 43 kDa band [66].

### Other tumor-type cell lines

The *DAG1* gene has been found to be downregulated at the mRNA level in cell lines derived from tumors of the hematopoietic system. Also, decreased expression levels were observed in the HL-60 and Kasumi-1 leukemia-derived cell lines of α-DG core protein (by ‒ 1.28- and ‒ 2.00-fold, respectively), and especially of β-DG (by ‒ 6.25- and ‒ 2.17-fold), correlating with an underexpression of *DAG1* mRNA (by ‒ 2.50- and ‒ 1.49-fold) [77, 85]. In parallel, it was reported that αDG was hypoglycosylated (by ~‒ 20- and ~‒ 9-fold, respectively) in both cell lines as compared to control samples from healthy individuals. Regarding β-DG, and in keeping with samples from patients with AML, it was noticeable that in these cell lines β-DG was found to translocate from the cytoplasm to the nucleus, where it was detectably phosphorylated [77].

In the melanoma cell lines BLM, M14 and IF6, glycosylation of α-DG was reported to be nearly absent [108]. Higher levels of β-DG were detected, however, in the non-metastatic melanoma IF6 cell line, whereas it was found at levels comparable to normal human melanocytes in the other two cell lines [108]. Yet, statistical analyses were not provided. Levels of α-DG matriglycan have also been found nearly undetectable in cell lines derived from human rhabdomyosarcomas [96] and lung cancer [69]. In the latter, as it was the case for other epithelial cancer cell lines (of breast or cervical origin), it was determined that, despite α-DG core protein was detectably expressed, they lacked a functional, laminin-binding α-DG.

Summarizing results above, α-DG has been found deficiently glycosylated in a wide variety of human tumor samples (Table 1) and cancer-derived cell lines (Table 2) of the digestive, reproductive, urinary, nervous, respiratory and musculoskeletal systems, in addition to breast cancer, leukemia and melanoma. These observations were accompanied by a downregulation of *DAG1* mRNA and/or α-DG (core) protein in a limited number of reports, and in conjunction with underexpression of β-DG protein in most cases. The implications of these findings in the light of both α-DG and β-DG involvement in tumorigenesis are discussed below.

## Alterations in the expression of dystroglycanopathy-associated genes and proteins in human tumors

**Fig. 2:**
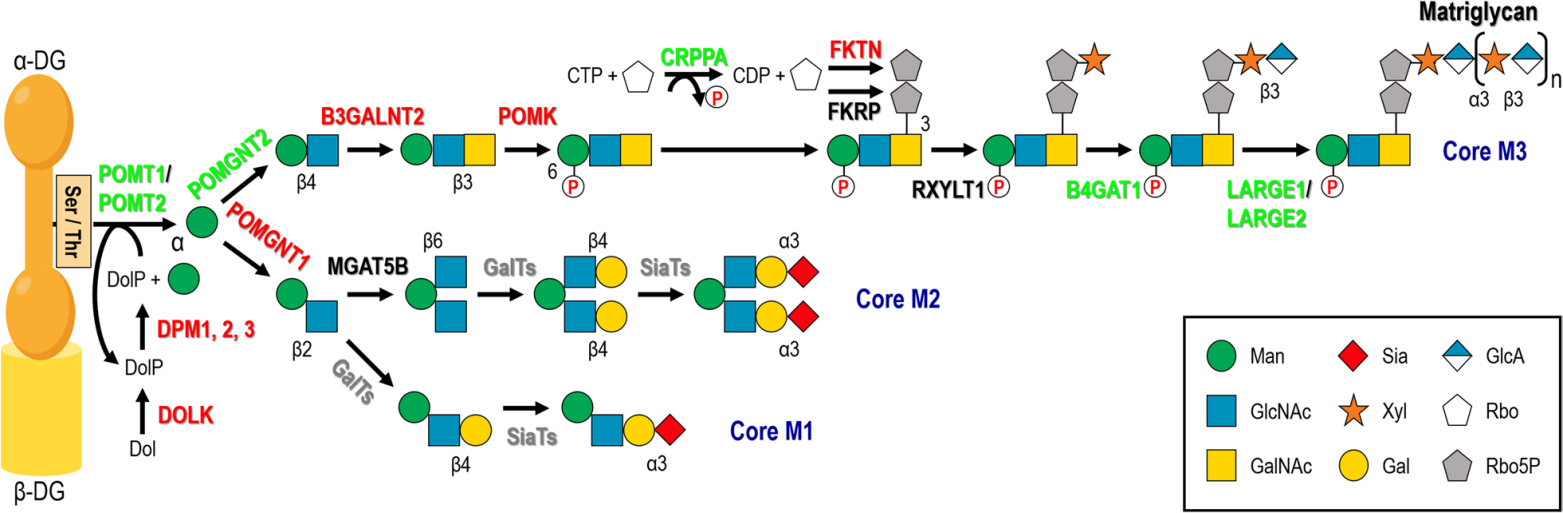
Alterations in the expression levels of dystroglycanopathy-associated genes in human tumor cells. The α-DG *O-*mannosyl glycosylation pathway is depicted, including branches leading to the synthesis of core M1, M2 and M3 glycans. The structure responsible for α-DG interaction with its ECM ligands, matriglycan, is synthesized on the core M3 glycan. Genes and their encoded proteins found to be mostly overexpressed in human solid tumors and cancer-derived established cell lines are indicated in red, and those found in most cases underexpressed are shown in green on their corresponding reactions. GalTs, galactosyltransferases; SiaTs, sialyltransferases

An altered expression at the mRNA and/or protein level(s) of genes associated with dystroglycanopathies, responsible for α-DG glycosylation, has also been evidenced in a variety of human primary tumors. The steps at which these genes and their protein products act in the α-DG *O*-mannosyl glycosylation pathway can be seen in Fig. 2. Alterations in their expression reported so far in human tumors are collected in Table 3.

**Table 3:** Alterations in the expression of dystroglycanopathy-associated genes and proteins in human tumors

Gene	Alteration	Levels	Tumor type	References
*POMT1*	mRNA	Underexpressed	Kidney	[76]
		Overexpressed	Acute myeloid leukemia (AML)	[77]
*POMT2*	mRNA	Underexpressed	Kidney	[76]
			Breast	[111]
		Overexpressed	AML	[77]
*DOLK*	mRNA	Overexpressed	Breast	[112]
*DPM1*	mRNA	Overexpressed	Head and neck squamous cells	[113]
			Esophagus	
			Colon	
			Rectum	
			Liver	
	Protein	Overexpressed	Liver	[113]
*DPM2*	mRNA	Overexpressed	Head and neck squamous cells	[113]
			Esophagus	
			Colon	
			Rectum	
			Liver	
	Protein	Overexpressed	Liver	[113]
*DPM3*	mRNA	Overexpressed	Esophagus	[113]
			Liver	
			Breast	
			Glioblastoma	
	Protein	Overexpressed	Liver	[113]
*POMGNT1*	mRNA	Overexpressed	Kidney	[76]
			Glioma	[114]
			AML	[77]
	Protein	Overexpressed	Glioma	[114, 115]
*POMGNT2*	mRNA	Underexpressed	Kidney	[76]
			Melanoma, uveal	[116]
*B3GALNT2*	mRNA	Underexpressed	Kidney	[76]
			Rhabdomyosarcoma	[96]
		Overexpressed	Liver	[117]
			Breast	[118]
	Protein	Overexpressed	Liver	[117]
*POMK*	mRNA	Underexpressed	Kidney	[76]
		Overexpressed	Breast	[119]
	Protein	Overexpressed	Breast	[119]
*CRPPA*	mRNA	Underexpressed	Kidney	[76, 120]
*FKTN*	mRNA	Overexpressed	Stomach	[121]
			Liver	[122, 123]
			AML	[77]
	Protein	Overexpressed	Stomach	[121]
*FKRP*	mRNA	Underexpressed	Kidney	[76]
			AML	[77]
*B4GAT1*	mRNA	Underexpressed	Pancreas	[124]
		Overexpressed	Kidney	[76]
*LARGE1*	mRNA	Underexpressed	Colon	[106]
			Prostate	[106]
			Kidney	[76, 106]
			Urinary bladder	[106]
			Breast	
			Glioma	
			AML	[77]
			Melanoma, skin	[106]
			Rhabdomyosarcoma	[96]
			Lung	[125]
		Overexpressed	Head and neck sqamous cells	[126]
			Liver	[127]
	Protein	Underexpressed	Oral squamous cells	[81]
*LARGE2*	mRNA	Underexpressed	Prostate	[87]
			Kidney	[76]
		Overexpressed	Colorectal	[128]

The table compiles all alterations reported so far in human tumors in the expression of dystroglycanopathy-associated genes at the mRNA and/or protein levels. Tumor types and references reporting these results are also indicated

The expression of *POMT1* and *POMT2* genes, which encode protein *O-*mannosyltransferases 1 and 2, two enzymes that act by forming a heterocomplex to carry out the first step in the *O*-mannosyl glycosylation of α-DG using dolichol-phosphate-mannose as a donor [129, 130], has been analyzed in clear cell carcinoma biopsy samples, with the result that both genes were underexpressed (by ‒ 1.82- and ‒ 1.27-fold, respectively) [76]. Also, *POMT1* and *POMT2* mRNA levels were determined in primary blasts derived from patients with AML. The results obtained revealed that in AML samples these genes were overexpressed (by 1.87- and 1.52-fold, respectively) in comparison to healthy individuals [77]. Additionally, the *POMT2* mRNA was underexpressed in breast tumor samples, which correlated with a reduced relapse-free survival and increased tumor aggressiveness [111].

The *DOLK* gene, encoding the enzyme dolichol kinase, has been found overexpressed (by 5.42-fold) at the mRNA level in breast cancer samples, especially from patients with triple-negative breast cancer (TNBC) [112], as seen in data available in the Gene Expression Omnibus (GEO) database (https://www.ncbi.nlm.nih.gov/geo), accession no. GSE38959. In addition, the expression of genes *DPM1*, *2* and *3*, encoding the three subunits of dolichol-phosphate mannosyltransferase complex, has been found altered in several tumor types. From several databases it has been determined that genes *DPM1* and *2* are overexpressed, at the mRNA level, in head and neck squamous cell carcinoma, and esophagus, colon, rectum and hepatic cancers (by 1.52- and 2.07-fold for *DPM1* and *DPM2*, respectively, in the latter), while *DPM3* is observed overexpressed in esophagus, liver (by 1.67-fold) and breast cancers, as well as in glioblastoma [113]. These three genes have been reported to be overexpressed also at the protein level in hepatic cancer, this being associated with clinical cancer stage, pathological tumor grade and a shorter patient survival [113].

The *POMGNT1* gene, encoding protein *O-*linked-mannose β-1,2-*N*-acetylglucosaminyltransferase 1, which is the next enzyme in acting after POMT1 and 2 by branching the α-DG *O*-glycosylation pathway in competence with the *POMGNT2* gene product enzyme [131] (Fig. 2), has been found overexpressed in grade III and IV gliomas, this correlating with significantly lower survival in patients with glioblastoma (grade IV glioma) [114]. Overexpression of the POMGNT1 protein has been evidenced as well in these types of advanced gliomas, its levels increasing in a correlated fashion with the degree of tumor progression (till ~ 15-fold), as a difference with grade I and II gliomas where expression of this protein was undetectable [114, 115]. Furthermore, and as it was the case for *POMT1*/*2*, the *POMGNT1* gene was found upregulated at the mRNA level in renal cell carcinoma (by 1.20-fold) [76] and primary blasts derived from patients with AML (by 1.85-fold) [77].

The expression of *POMGNT2* gene, encoding protein *O*-mannosyl β-1,4-*N*-acetylglucosaminyltransferase 2, has been found reduced (by ‒ 2.22-fold) in kidney cancer [76]. Also, this gene’s downregulation has been detected altered as part of a ten-gene signature in uveal melanoma (an intraocular primary cancer) [116]. The *POMGNT2* gene was found underexpressed at the mRNA level, alike six of the other genes, whereas the remaining three genes were found overexpressed. This expression pattern has been found in patients with high-risk uveal melanoma and has been correlated with a low survival and a higher probability of developing metastasis [116]. Alterations in the expression of the *B3GALNT2* gene, encoding β-1,3-*N*-acetylgalactosaminyltransferase 2, have been reported in cancer, being upregulated both its mRNA and protein product (the latter by 1.31-fold) in samples of hepatocellular carcinoma, and its levels directly correlating with tumor size and with a lower survival of patients with this tumor type [117]. Furthermore, the *B3GALNT2* mRNA has been found overexpressed (by 2.56-fold) in breast cancer samples, especially from patients with TNBC [118], although *B3GALNT2* was detectably downregulated in rhabdomyosarcoma [96] and kidney carcinoma (by ‒ 1.82-fold) [76] biopsy samples.

Increased levels (by 1.56-fold) of the mRNA of the *POMK* gene (formerly *SGK196*), encoding protein *O*-mannose kinase, have also been found in different types of breast cancer, compared to healthy breast tissue, by analyzing the data compiled in the Gene Expression Profiling Interactive Analysis (GEPIA; http://gepia.cancer-pku.cn) and Oncomine (https://www.oncomine.org/resource/login.html) microarray databases [119]. Overexpression of this gene at the protein level has also been detected in breast cancer by immunohistochemistry, with POMK being found both *N*-glycosylated (in a process regulated by ribophorin 1, or RPN1) and lacking *N*-glycosylation in both tumor and healthy tissues. It was determined, however, that in basal-type breast cancer (BLBC) POMK is only present in its *N*-glycosylated form, its overexpression being related with a better relapse-free survival from this subtype of tumor [119]. Also, it was noticed that a lower expression of *N*-glycosylated POMK promoted an increased phosphorylation of the AKT serine/threonine kinase, with ensuing activation of the PI3K/AKT/glycogen synthase kinase-3β (GSK-3β)/Snail signaling pathway, involved in metastasis, while the expression of α-DG core protein remained essentially unaltered [119]. Regarding kidney cancer, *POMK* has been found slightly downregulated (by ‒ 1.23-fold) in human biopsy samples [76].

An upregulation at the mRNA or protein level of the gene encoding fukutin (*FKTN*) has been found to be related to the tissue of origin or the progression of tumors in patients with stomach cancer [121]. This gene has been found overexpressed at the mRNA level also in hepatocellular carcinoma, together with other five genes related to lactate metabolism, in a fashion associated with pathological grade, clinical stage and vascular invasion, as well as with overall and median survival of patients [122, 123], and in primary blasts derived from patients with AML (by 2.22-fold) [77]. The *B4GAT1* gene (formerly *B3GNT1*) has been detected underexpressed at the mRNA level in pancreatic ductal adenocarcinoma, this being associated with a poor patient prognosis [124]. In contrast, this gene was overexpressed in renal cell carcinoma (by 1.26-fold) [76]. Reduced mRNA levels of the genes *FKRP* and *LARGE1*, encoding fukutin-related protein and LARGE xylosyl- and glucuronyltransferase 1, respectively, have been reported in AML primary blasts (by ‒ 1.89 and ‒ 3.03, respectively), at variance with genes *POMT1*, *POMT2* and *POMGNT1*, which were found overexpressed in cells of this tumor type (see above) [77]. Separately, *LARGE1* mRNA expression data, retrieved from the Oncomine database, have been compared between cancerous and healthy tissues of different types. A deficient expression of *LARGE1* was detected in colon adenocarcinoma (by ‒ 1.30-fold), prostate cancer (by ‒ 1.61-fold), infiltrating urinary-bladder cancer (by ‒ 1.85-fold), breast carcinoma (by ‒ 1.20-fold), glioma (by ‒ 2.63-fold) and melanoma (by ‒ 1.45-fold) [106], as well as in renal cell carcinoma (clear cells) (by ‒ 1.72 or ‒ 1.45-fold) [76, 106], rhabdomyosarcoma [96] and non-small-cell lung cancer [125]. Interestingly, the *LARGE1* gene was originally identified at a chromosomal region frequently deleted in human meningiomas [132] that, since it falls within a common fragile site, it is prone to suffer breaks and deletions in other cancer types [133]. Consequently, a loss of somatic copies of the *LARGE1* gene has been reported in patients with primary glioblastoma, this being related to an increase of Ki-67^+^ cells and a shortened survival [134]. On the other hand, a significant overexpression of *LARGE1* mRNA was observed in squamous cell carcinoma of head and neck [126] and hepatic cancer (by ~ 5-fold) [127], being associated in the latter with a low patient survival. However, it should be noted that an absent expression of LARGE1 protein has been found in tongue squamous cell carcinoma, which statistically correlated with a reduction in glycosylated α-DG levels in this pathologic type of cancer [81]. Finally, The Cancer Genome Atlas (TCGA) database (https://portal.gdc.cancer.gov) was used to identify possible changes in the mRNA expression of genes involved in α-DG glycosylation in colon and kidney carcinomas from different patients [76]. At variance with *LARGE1*, data collected in this database on advanced colorectal cancer (CRC) samples reflected elevated levels of *LARGE2* mRNA. This is a *LARGE1* paralog so far not found mutated in any DGP cases, but whose encoded enzyme catalyzes the same reaction (see Fig. 2) and whose involvement in α-DG glycosylation (i.e., matriglycan synthesis) is also crucial [135]. Upregulation of *LARGE2* has also been found in organoids derived from patients with primary CRC and liver metastatic tissue samples, although the expression increase was not significant in the latter [128]. On the other hand, a significant reduction in the mRNA levels of *CRPPA* (formerly *ISPD* [35]), encoding *D-*ribitol-5-phosphate cytidylyltransferase [76, 120], and *LARGE2* genes (by ‒ 1.39- and ‒ 5.88-fold, respectively), was evidenced in patients with renal cell carcinoma, which was associated with an increased mortality [76]. The reason for this discrepancy between kidney and CRC data obeys to the CRC metastatic status in the context of α-DG reglycosylation in metastatic cells at their new tissue environment, as discussed below. Decreased expression of *LARGE2* (by ‒ 3.15-fold) has also been reported in prostate cancer [87].

## Alterations in the expression of dystroglycanopathy-associated genes and proteins in human cancer cell lines

The expression of genes and proteins associated with DGPs has also been analyzed in a wide variety of human tumor-derived cell lines. Table 4 enlists all the alterations so far detected, at the mRNA and/or protein level(s).

**Table 4:** Alterations in the expression of dystroglycanopathy-associated genes and proteins in human cancer cell lines

Gene	Alteration	Levels	Tumor type	Cell line	References
*POMT1*	mRNA	Underexpressed	Colon	HCT116	[136]
			Acute promyelocytic leukemia (APL)	HL-60	[77]
			Acute myeloid leukemia (AML)	Kasumi-1	
*POMT2*	mRNA	Underexpressed	Stomach	Kato III	[137]
			Breast	MCF7	[111]
				MDA-MB-231, -453	
				T47D	
			APL	HL-60	[77]
			AML	Kasumi-1	
	Protein	Underexpressed	Stomach	Kato III	[137]
*DPM1*	mRNA	Overexpressed	Liver	Hep G2	[113]
				SMMC-7721	
*DPM2*	mRNA	Overexpressed	Liver	SMMC-7721	[113]
*DPM3*	mRNA	Overexpressed	Liver	SMMC-7721	[113]
*POMGNT1*	mRNA	Underexpressed	APL	HL-60	[77]
			AML	Kasumi-1	
*B3GALNT2*	mRNA	Underexpressed	Liver	BEL-7405	[117]
		Overexpressed	Liver	Huh7	
				Hepa 3B	
				Hep G2	
				SMMC-7721	
				BEL-7402, 7404	
			Breast	MDA-MB-453	[118]
				HCC1143, 1395, 1599	
				T47D	
				BT-20	
				ZR-75-1	
				OCUB-F	
	Protein	Overexpressed	Liver	Huh7	[117]
				Hepa 3B	
				Hep G2	
				SMMC-7721	
				BEL-7402, -7404, -7405	
*FKTN*	mRNA	Underexpressed	APL	HL-60	[77]
			AML	Kasumi-1	
		Overexpressed	Stomach	MKN-1, 45	[121]
	Protein	Underexpressed	Cervix	HeLa	[138]
		Overexpressed	Stomach	MKN-1, 45	[121]
				HSC-57	
*FKRP*	mRNA	Underexpressed	APL	HL-60	[77]
			AML	Kasumi-1	
*B4GAT1*	mRNA	Underexpressed	Prostate	PC3	[105]
				PC3-L	
				LNCaP	
			Breast	MDA-MB-231, -435	
*LARGE1*	mRNA	Overexpressed	Liver	Huh7	[127]
				Hep G2	
				SMMC-7721	
		Underexpressed	Oral squamous cells	SCC-4	[81]
				CAL27	
			Pancreas	PANC-1	[87]
				BxPC-3	
			Prostate	PC3	[87]
			Cervix	HeLa	[69]
			Breast	MDA-MB-231, -436, 468	[69, 87, 106]
				HCC38, 70, 1143, 1500, 1937	[106]
				SUM149-PT, 159PT	
			Glioblastoma	U87MG	[106]
				U251MG	
				LN-18	
			APL	HL-60	[77]
			AML	Kasumi-1	
			Rhabdomyosarcoma	Rh18	[96]
			Lung	H1299	[69]
				H2030	
	Protein	Underexpressed	Breast	MDA-MB-231	[107]
			Glioblastoma	U87MG	[106]
				U251MG	
				LN-18	[106, 107]
*LARGE2*	mRNA	Underexpressed	Pancreas	PANC-1	[87]
				BxPC-3	
			Colon	HT-29	[87]
				Colo205	
				RKO	
			Prostate	TEM4-18	[87, 103]
			Kidney	A498	[103]
			Breast	MDA-MB-231	[87, 103]
				ZR-75-1	[87]
		Overexpressed	Colon	SW480, 620	[128]
				LS174T	

The table compiles all alterations reported so far in human cancer cell lines in the expression of dystroglycanopathy-associated genes at the mRNA and/or protein levels. The tissue of origin of each cell line and references reporting these results are also indicated

The *POMT1* mRNA has been reported to be downregulated as a consequence of promoter hypermethylation in the HCT116 cell line of colon cancer, whereas normal colon mucosa cells lacked *POMT1* hypermethylation [136]. The *POMT2* gene (but not *POMT1*) has been found underexpressed in the breast cancer cell lines MCF7, MDA-MB-231, MDA-MB-253 and T47D, in keeping with observations in samples from patients with breast cancer [111]. In addition, *POMT2* has been found downregulated at the mRNA and protein levels in the undifferentiated gastric carcinoma cell line Kato III, in a coherent fashion with reduced global protein *O-*mannosyl glycosylation in this cell line and in human gastric cancers in general [137]. The mRNA levels of *POMT1* and *POMT2* genes have also been analyzed, together with *POMGNT1*, in the HL-60 and Kasumi-1 leukemia cell lines. These three genes were concluded to exhibit a reduced expression in both cell lines (by ‒ 3.80-, ‒ 7.14- and ‒ 4.00-fold, respectively, for HL-60, and by ‒ 1.43, ‒ 3.33- and ‒ 2.70-fold, respectively, for Kasumi-1), although at a difference with samples from patients with AML, where the three were detected to be overexpressed [77].

Coherently with results reported in patients with liver cancer, *DPM1*, *2* and *3* genes are overexpressed (by 4.00-, 1.51- and 1.47-fold), at the mRNA level in the SMMC-7721 cell line. Also, and at variance with *DPM2* and *3*, the *DPM1* mRNA was upregulated (by 1.50-fold) in the Hep G2 hepatic cancer cell line, which suggests that *DPM1* could be a potential biomarker with survival prognostic value in patients with hepatocellular carcinoma [113].

Regarding *B3GALNT2*, it has been found upregulated at both mRNA (from 1.08- to 2.85-fold) and protein (from 1.51- till 2.97-fold) levels in all hepatocellular carcinoma cell lines analyzed, with the sole exception of BEL-7405, where a reduced expression (by ‒2.56-fold) of *B3GALNT2* was observed at the mRNA, but not at the protein level [117]. Additionally, an overexpression of this gene’s mRNA has been evidenced in all (a total of eight) breast cancer cell lines studied (see Table 4) [118].

A reduced expression of the *FKTN* gene has been observed at the mRNA level in the HL-60 and Kasumi-1 leukemia cell lines (by ~‒4- and ~‒10-fold, respectively), again in contrast to the overexpression exhibited by patients with AML [77]. Undetectable levels of its protein product, fukutin, have also been reported in the HeLa cervical cancer cell line [138]. On the other hand, the *FKTN* mRNA was found upregulated in the stomach cancer cell lines MKN-1 and MKN-45, and at the protein level additionally in the HSC-57 cell line, which was consistent with the overexpression also reported in samples from patients with stomach cancer [121]. Alike *FKTN*, the *FKRP* mRNA was found underexpressed in the HL-60 and Kasumi-1 cell lines (by ‒ 6.67- and ‒ 2.50-fold, respectively), which was in keeping with the results from patients with AML [77].

The mRNA levels of the *B4GAT1* gene have been found to be decreased or undetectable in cell lines derived from prostate cancer, including PC3, PC3-L and LNCaP, and from breast cancer, such as MDA-MB-231 and MDA-MB-435 [105]. Its encoded protein, β-1,4-glucuronyltransferase 1 (B4GAT1), acts sequentially with LARGE1 in the synthesis of matriglycan on α-DG, i.e., the last steps of its *O*-mannosyl glycosylation pathway (Fig. 2). In a coherent fashion, *LARGE1* mRNA levels were very low or undetectable in the PC3 prostate cancer line [87] and in a variety (a total of ten) of breast cancer cell lines examined, including MDA-MB-231 [69, 87, 106]. Additionally, the *LARGE1* mRNA was found undetectable in the SCC-4 and CAL27 cell lines of oral squamous cell carcinoma [81], in the PANC-1 and BxPC-3 cell lines of pancreatic cancer [87] and in the HeLa cell line of cervical cancer [69] (Table 4). Other instances of *LARGE1* mRNA downregulation were found in the glioblastoma cell lines U87MG, U251MG and LN-18 (by ‒ 6.67-fold) [106], in the leukemia cell lines HL-60 and Kasumi-1 (by ‒ 3.33-fold) [77], in the rhabdomyosarcoma cell lines Rh18 and others [96], and in the lung cancer cell lines H1299 and H2030 where it was almost undetectable [69]. Also consistently, its LARGE1 protein product was virtually absent in the MDA-MB-231 breast cancer cell line and in the three glioblastoma cell lines just cited above [106, 107]. However, overexpression of the *LARGE1* mRNA has been detected in the hepatic cancer cell lines Huh7 (by 6.20-fold), Hep G2 (by 4.40-fold) and SMMC-7721 (by 7.50-fold), in correlation with cell growth, proliferation, migration, invasiveness and cell cycle [127]. Finally, decreased mRNA levels of the *LARGE2* gene have been detected in the PANC-1 and BxPC-3 cell lines of pancreatic cancer, TEM4-18 of prostate cancer, A498 of renal cell carcinoma, and MDA-MB-231 and ZR-75-1 of breast cancer [87, 103], whereas in colon cancer cell lines they were highly variable [87, 128].

Trying to summarize results above, if only human solid tumors and their derived cell lines are taken into account, it can be concluded that genes *DOLK*, *DPM1*/*2*/*3*, *POMGNT1*, *B3GALNT2*, *POMK* and *FKTN* and/or their encoded proteins, all of which (except fukutin) act at early steps of the α-DG *O*-mannosyl glycosylation pathway rendering core M1-M3 glycans (see Fig. 2), are in general upregulated (Tables 3 and 4). However, and in an opposite fashion, *POMGNT2*, *CRPPA*, *B4GAT1*, *LARGE1* and *LARGE2* are consistently downregulated in human tumors and derived cell lines, with very few exceptions (Tables 3 and 4), all of which are more directly involved than the set of enzymes above in the synthesis of matriglycan on the phosphorylated α-DG core M3 glycan (Fig. 2). It seems thus reasonable that, since this structure is crucial for α-DG function, their underexpression should lead to a loss of the cells’ ECM-binding ability with ensuing enhanced cell proliferation and migratory abilities, this bringing into light their normal role as tumor suppressors, as documented below. Conversely, a pro-oncogenic role of the above overexpressed genes (*DOLK*, *DPM1*/*2*/*3*, *POMGNT1*, *B3GALNT2*, *POMK* and *FKTN*), acting at earlier steps of the α-DG glycosylation process, is apparent instead, exerted (among other mechanisms) through the modulation of intracellular signaling pathways, as also explained in the next sections. Regarding leukemia, controversial data have been provided between AML tumors and cell lines for mRNA levels of most of DGP-associated genes, whose expression additionally has not been addressed at the protein level, which precludes to draw consistent conclusions and calls for further investigations.

## Loss of dystroglycan binding to laminin, cell binding to the extracellular matrix and cell-cell adhesion

Considering data obtained so far on alterations in the glycosylation and/or expression of DG and DGP-associated genes, responsible for α-DG glycosylation, a correlation has been underlined between such alterations, especially hypoglycosylation of α-DG, and the progression (tumor grade and/or stage), severity and poor prognosis of cancer. Such relationship has been experimentally documented, as mentioned above, in gastric [83], pancreatic [84], colon [85, 86], prostatic [87], cervical, vulvar [90], renal [91] and mammary [85] cancers, as well as in gliomas [95] and neuroblastomas [93]. In this and following sections possible mechanisms accounting for the involvement of DG (α and β subunits) in cancer pathogenesis are addressed.

In the light of the effect brought about by α-DG hypoglycosylation on tumorigenesis, analyses have been made on laminin binding in a number of human established cancer cell lines, mainly derived from prostate carcinomas. All the prostate cancer cell lines studied so far that exhibited hypoglycosylated α-DG in terms of matriglycan levels (Table 2) had concomitantly lost their ability to bind laminin, and the same has been evidenced for cervical cancer, glioblastoma, lung cancer and in the vast majority of breast cancer lines. In the latter, a large number of different cell lines have been examined, and a correlation was found between positive α-DG glycosylation and ability to bind laminin and, conversely, between hypoglycosylated α-DG and loss of this property [67, 69, 87, 105, 106], although with a few exceptions [106]. It was found that in those cell lines bearing a positively-glycosylated α-DG, such as 22Rv1, PC3 and its PC3-H and PC3-E+ derived, less-aggressive subpopulations, the latter two exhibiting a heavily-glycosylated α-DG, a high capacity of binding exogenous laminin was evidenced [87, 105]. By contrast, LNCaP and two PC3-derived cell lines lacking matriglycan, namely PC3-L and TEM4-18 (see Table 2), were unable to bind exogenous laminin and simultaneously exhibited a higher migration ability and aggressiveness than their parental, PC3 cell line. An inverse correlation was thus established between the expression of laminin-binding glycans and the migratory and invasive potential, and hence the aggressiveness and malignancy, of prostate carcinoma cells [87, 105].

It has also been evidenced that the higher or lower α-DG matriglycan levels in the above prostate cell lines (and especially TEM4-18) are directly correlated with the mRNA levels of the *LARGE2* gene [87]. Further, *LARGE2* expression inversely correlated with tumor progression, from a localized to an invasive stage, in a tissue cDNA microarray organized according to prostate cancer stage. It followed that LARGE2 functionally glycosylated α-DG, thereby reducing the cells’ invasive and proliferative potential. At variance, in the PC3-L cell line it was documented that its virtual lack of laminin-binding glycans and enhanced cell migration was associated with a reduced expression of the *B4GAT1* gene (see Table 4), whose protein product was evidenced to form a complex with LARGE1 or LARGE2 to positively modulate their function [105]. These studies have unveiled a new role of glycan-dependent interaction between the cells and the basement membrane in tumor suppression and its control by B4GAT1 and LARGE1 or LARGE2 depending on the tissue. Furthermore, individual downregulation of any of their encoding genes (by means of siRNA knockdown), and also of *POMT1* or *DAG1* itself, led to an increase of the levels of phosphorylated AKT and ERK in PC3 cells [105]. This indicated that the antimigratory activity elicited by α-DG binding to its ECM ligands was exerted by counteracting the activation of ERK and AKT taking place in response to integrin engagement on laminin [105, 139]. Also, this connects with the role of β-DG as a multifunctional adaptor or scaffold capable of physically interacting, by means of its cytoplasmic C-terminus, with Grb2 and c-Src, and associate with components of the ERK/MAPK (such as MEK and ERK) and PI3K/AKT cascades, thereby negatively modulating these pathways [27, 28, 51–53, 102, 140, 141], as addressed below.

In human rhabdomyosarcoma biopsies [96] and a number of epithelial cancer-derived cell lines [69, 81], an anomalous silencing of the *LARGE1* gene has been determined to be a primary cause of loss of laminin binding by DG. This defect was attributed to an epigenetic phenomenon in the HeLa (cervix), MDA-MB-231 (breast) and H1299 and H2030 (lung) cell lines, where in all cases the *LARGE1* promoter was found hypermethylated [69], as it also occurred in the SCC-4 and CAL27 (tongue squamous) cell lines [81]. In this context, when treating all these 6 cell lines with a demethylating agent, alone or in combination with a histone deacetylase inhibitor, *LARGE1* expression and (partially) α-DG matriglycan levels were recovered, this rescuing their ability to bind laminin and decreasing their migratory ability [69, 81]. However, the loss of laminin-binding capacity was not attributable solely to the deficiency in *LARGE1* expression, since an underexpression of other genes involved in α-DG glycosylation has been documented in some of these cell lines, namely the mRNAs of genes *POMT2*, this likely due to epigenetic regulation exerted by the histone methyltransferase EZH2 [111], *B4GAT1* [105] and *LARGE2* in MDA-MB-231 cells [87, 103] and fukutin protein in HeLa cells [138] (Table 4). In this light, fukutin appears to act inside the nucleus of HeLa and ZR-75-1 cells to suppress cell proliferation (determined as the number of Ki-67^+^ cells), likely by inhibiting activation of the mitogenic transcription factor, c-Jun [138, 142]. By contrast, a role of fukutin in the nucleus to activate cell proliferation has been reported in the 1321N1 astrocytoma cell line, this suggesting that fukutin could act in a different fashion depending on the cell type [143].

Additionally, studies have been made on the expression of epithelial cadherin (E-cadherin) gene, *CDH1*, in a number of tissues and tumor cell lines with altered DG expression and/or glycosylation. E-cadherin is crucial in the regulation of motility and proliferation of epithelial cells, exhibiting a potent suppressor of invasiveness role [144], and it is known that E-cadherin is lost during metastatic spread, a process resembling in many aspects the EMT normally occurring during embryonic development and involving changes in cell-cell and cell-ECM adhesion [28, 63, 145]. The EMT process allows epithelial cells to transform into mesenchymal cells and involves E-cadherin downregulation, so cells can detach from laminin, migrate to other organs and eventually metastasize [57, 103]. These events have also been reported in human cancer stem cells derived from mesenchymal-like glioblastomas [102]. In this regard, a correlation has been found by flow cytometry between functional α-DG glycosylation (i.e., matriglycan levels) and E-cadherin expression (or simultaneous lack of both molecular properties) in several established cell lines derived from a variety of human tumor types, including colon, prostate, renal and breast cancers, as well as epidermoid carcinoma, although this was not always the case [103].

In samples of patients with esophageal cancer a loss of α-DG matriglycan has been detected together with a downregulation of β-DG (Table 1) and E-cadherin expression at the protein level, with these two molecules showing a similar immunolocalization pattern in the intercellular and basement membrane regions in esophageal carcinoma samples [82]. In a similar fashion, in the FLO-1 cell line both the glycosylation of α-DG and expression of β-DG (Table 2) together with E-cadherin dropped steadily under culture conditions, until becoming negligible or undetectable, whereas mRNA levels remained intact. In human primary gastric carcinoma samples, as well as in the undifferentiated cell line Kato III, low levels of protein *O*-mannosyl glycosylation are found overall, and especially of E-cadherin, which is both detectably underexpressed and poorly *O*-glycosylated, this leading to a partial cytoplasmic mislocalization of the latter with loss of cell-cell adhesion capability [137]. This occurred in an associated fashion with increased levels of *MGAT5* mRNA, encoding the *N*-acetylglucosaminyltransferase named GnT-Va, and reduced levels of *POMT2* mRNA and protein. These and other findings support the notion that a coordinated interplay exists between *O*-mannosyl glycosylation and *N-*glycosylation positively and negatively modulating, respectively, E-cadherin function, and that loss of the first in favor of the second could lead to loss of E-cadherin suppressive functions in cancer, thus contributing to tumor progression and metastasis [137].

Separately, studies of the expression of E-cadherin and α-DG glycosylation (matriglycan levels) have been conducted on breast cancer cell lines, where it was found that lack of polarized cell growth only took place in those cell lines exhibiting negative α-DG glycosylation and/or expression together with negative E-cadherin levels [67]. This was attributable to epithelial-cell polarity requiring both cell adhesion to ECM (mediated by glycosylated α-DG binding to laminin), and cell-cell interaction (mediated by E-cadherin) [146]. It was also noticed that in the T4-2 cell line, which exhibited low levels of both α-DG matriglycan and β-DG protein (Table 2), the epidermal growth factor receptor (EGFR) and integrin β1 were both overexpressed. When *DAG1* (cDNA) was overexpressed in T4-2 cells, cell rounding (reflecting cytoskeletal changes) was elicited by laminin, cell polarity was restored and their tumorigenic potential reduced by increasing the levels of PTEN [67], a well known phosphatase acting as a tumor suppressor by inhibiting AKT/GSK-3β/Snail signaling [147].

α-DG expression and its functional glycosylation have also been detected on tumorigenic glioma stem cells (GSCs) residing in the perivascular bed of the tumor and in the most aggressive mesenchymal-like (MES-like) GBM tumor compartment [102]. Within the high cellular heterogeneity exhibited by GBM, the self-renewing GSCs are MES-like cells in which DG acts to maintain such status via a tight control of MAPK activation [102, 148]. In this context, antibody-based blockade of α-DG induces a robust ERK-mediated differentiation leading to reduced GSC potential [102]. Laminin-integrin interactions can activate ERK, but only in cells expressing predominantly integrin α6A with an intact cytoplasmic tail, and not integrin α6B [149]. However, in GSCs integrin α6B and the mesenchymal markers neural cadherin (N-cadherin) and Slug are predominantly expressed, while integrin α6A and the epithelial marker E-cadherin are essentially absent [102], as it was the case in a previous study in breast cancer where integrin α6A was expressed in epithelial tumor cells and integrin α6B in mesenchymal tumor cells [150]. These data suggest that the co-expression of DG and integrin α6B in MES-type tumor cells contributes to tumor maintenance, promoting its aggressiveness and malignancy. Loss of DG matriglycan and E-cadherin expression during the EMT process taking place at the primary tumor site, promoting dissemination and invasiveness of tumor cells therefrom and hence their metastatic spread, is followed by mesenchymal-epithelial transition (MET) occurring at the secondary site [151]. This process has been proposed to involve recovery of α-DG functional glycosylation together with E-cadherin expression during metastatic colonization, this favoring the adhesion of the growing secondary tumor to the ECM of the new tissue [96, 151].

Finally, in breast cancer, although a correlation between α-DG hypoglycosylation and loss of their ability to laminin anchoring has been demonstrated in many cell lines [67, 69, 87, 105, 106], a few exceptions exist of cell lines which, despite exhibiting hypoglycosylated α-DG, are yet able to adhere to laminin [106], this requiring further investigation. Recently, it has been shown that the addition of ribitol to breast cancer cells (MCF7 and T47D cell lines) enhances matriglycan synthesis on α-DG, albeit without decreasing their proliferation rate, migration or growth in Matrigel (i.e., invasiveness) [152]. It must be emphasized here that ribitol is intracellularly transformed into CDP-ribitol, this acting as a substrate for fukutin and FKRP in the synthesis of matriglycan on the α-DG core M3 glycan (see Fig. 2).

## Tumor-suppressing or pro-oncogenic role of dystroglycanopathy-associated proteins

In human solid tumors and derived cell lines, genes *POMT1*/*2*, *POMGNT2*, *CRPPA, B4GAT1*, *LARGE1/2* and/or their encoded proteins are consistently downregulated, with very few exceptions (Tables 3,  4). Since they are directly involved in the synthesis of the α-DG phosphorylated core M3 glycan or of matriglycan on the latter, and this structure is essential for α-DG function, their underexpression directly correlates with loss of α-DG ability to bind laminin and, presumably, also other ECM ligands. Consequently, a tumor-suppressing role has been explicitly proposed for *DAG1* and some of the DGP-associated genes [67, 71, 97, 105, 141]. This is at variance with *DOLK*, *DPM1/2/3*, *POMGNT1*, *B3GALNT2*, *POMK* and *FKTN*/fukutin, which have been found mostly upregulated at the mRNA and protein levels (Tables 3, 4), and hence can be presumed to exhibit a pro-oncogenic behavior. These proteins act at earlier steps of α-DG *O*-mannosyl glycosylation rendering core M1-M3 glycans, and their involvement in tumorigenesis should thus not be related to their catalytic activity itself. Indeed, the overexpression of none of these genes has ever been reported to result in α-DG hyperglycosylation (or α-DG core protein overexpression), and their tumorigenic action must thus be exerted instead through modulation of signal transduction or gene expression.

It has also been determined that cell lines lacking both α-DG matriglycan and E-cadherin expression, such as TEM4-18 (prostate), A498 (kidney) and MDA-MB-231 (breast), displayed a high mRNA expression of the gene encoding the EMT-promoting, zinc-finger transcription factor ZEB1 together with a downregulation of *CDH1* and *LARGE2* genes, as compared to the less-aggressive PC3-E+ (prostate) cell line, which exhibited both molecular phenotypes [103]. Human tumors were thus checked for expression of *ZEB1* and *LARGE2* genes through examination of clinical samples deposited in the TCGA database. It was found that mRNA levels of both genes inversely correlated between them in four types of cancer: prostate adenocarcinoma, clear cell renal cell carcinoma, invasive breast carcinoma and lung adenocarcinoma, in which DG expression and/or functional glycosylation were downregulated [103]. Experiments were then made in which the (artificial) overexpression of ZEB1 or its upstream effector Snail separately hindered in PC3-E + cells both E-cadherin and α-DG matriglycan levels, and also (albeit less importantly) β-DG expression. It was also evidenced that ZEB1 repressed *LARGE2* expression by directly binding to its promoter [103]. However, silencing (by siRNA transfection) of ZEB1 and/or Snail expression was not sufficient to rescue functional α-DG glycosylation, despite restoring mRNA expression of genes encoding LARGE2 and E-cadherin [103]. This could be attributed to the downregulation of *B4GAT1* and *LARGE1* genes existing in prostate cancer cell lines, as it was also the case for breast cancer cell lines (Table 4). In this context, and interestingly, *LARGE2* has been recently demonstrated to be a Wnt target gene in CRC cells. Here the tumor suppressor protein APC (a Wnt signaling pathway regulator) is frequently inactivated by mutation, which allows Wnt activation and the concomitant nuclear translocation of β-catenin [128, 153, 154]. This acts as a coactivator which associates inside the nucleus with the transcription factor TCF7L2 (also called TCF4), a Wnt effector that in turn binds to *LARGE2* intron 1 and activates its mRNA expression, with ensuing enhancement of LARGE2-dependent matriglycan synthesis on α-DG. This results in an increase of α-DG laminin-binding capacity and, associatedly, in a reduction of CRC cells migration and invasiveness [128]. α-DG glycosylation has also been described to interfere with these two cellular properties in renal and prostate cancers [87, 105], as pointed out above.

On the other hand, little is known on the mechanisms underlying the pro-oncogenic action of several DGP-associated proteins, since they have been documented experimentally so far in a limited number of instances of human tumor types and cancer-derived cell lines. In this light, POMGNT1 clearly exerts a pro-oncogenic role in glioblastoma, likely by inhibiting the intrinsic phosphatase activity of receptor-type protein tyrosine phosphatase β/ζ (RPTPβ/ζ) through its *O-*mannosyl glycosylation possibly mediated by both POMGNT1 and GnT-Vb, the latter being highly expressed in the brain [114, 155–157] and also through downregulation of RPTPβ/ζ protein expression mediated by POMGNT1 [114]. This negative modulation of RPTPβ/ζ results in activation of β-catenin derived from its increased tyrosine phosphorylation, with ensuing release of β-catenin from E-cadherin, its nuclear translocation and its promoted transcriptional activation of Wnt-target, EMT-promoting genes hindering cell-cell adhesion and promoting cell proliferation and invasiveness [114, 128, 145, 153, 158, 159]. Conversely, *POMGNT1* silencing decreases cell growth and invasion in human glioblastoma cell lines [114]. In this context, it has been recently found that *POMGNT1* knockout in the human HEK293T cell line also leads to a lower proliferation and migration rates, in a fashion associated with loss of cell-laminin interaction and increase of cell-cell adhesion mediated by N-cadherin [160]. This was attributable to an upregulation of this protein and its encoding mRNA (*CDH2* gene) found together with a quantitatively and qualitatively altered N*-*cadherin *N-*glycosylation, possibly related to a mildly-decreased *MGAT5* expression, encoding the GnT-Va enzyme. Also, a sustained activation of the ERK/MAPK pathway was demonstrated, reflected by increased levels of phosphorylated ERK1/2, p38 MAPK and MEK, together with overexpression of a set of EMT-promoting transcription factors, including Snail [160]. These and other molecular events indicative of the occurrence of an EMT-like transition were also evidenced in skin fibroblasts from a MEB patient, harboring a *POMGNT1* D179Rfs*11 mutation, this contributing to explain some of his/her clinical DGP symptoms. These results are in keeping with the link (i.e., inverse relationship) between classic *O-*mannosylation and *N-*glycosylation of E-cadherin pointed out above, with changes in the first impacting on the second, unveiled in human gastric carcinomas [137] and during mouse embryonic development [161].

Regarding POMK, although found overexpressed in breast cancer, a mechanism thought to play a protective role especially in BLBC, its *N-*glycosylation status appears to be more relevant than its expression levels for its tumor-suppressing (anti-invasion and anti-metastasis) capacity and hence for a better patient prognosis [119]. In this light, *N-*glycosylated POMK has been evidenced to interact with signaling proteins (other than α-DG) to inhibit the AKT/GSK-3β/Snail metastatic/EMT pathway in breast cancer cells [119] preventing (i) GSK-3β activation and ensuing β-catenin accumulation in the cytoplasm with subsequent nuclear translocation [153], and (ii) Snail stabilization promoting E-cadherin downregulation and adult EMT [103, 119]. Yet, a deficient *N-*glycosylation in breast tumor tissue remains to be evidenced. In an opposite fashion, B3GALNT2 appears to act in breast cancer in a secreted, *N*-glycosylated form by indirectly promoting FGF2-elicited angiogenesis mediated by GnT-Va [118, 162], and in hepatic cancer (uncharacterized whether *N*-glycosylated) by indirectly increasing the activity of macrophage migration inhibitory factor (MIF), a proinflammatory cytokine [117]. Finally, *FKTN*/fukutin mRNA and protein in gastric cancer [121] and *FKTN* mRNA in hepatic cancer [122, 123] have been found upregulated, fukutin presumably acting as a coactivator of the AP1 transcription factor (constituted by c-Fos and c-Jun), with ensuing upregulation of cyclin D1 expression and increase of cell proliferation [143]. Interestingly, fukutin has been shown to interact with POMGNT1 in the Golgi complex, and alterations in its expression have been found to modulate POMGNT1 activity [163].

## Intracellular modulation by β-dystroglycan of cell proliferation and invasiveness

As documented above, both DG expression (α and/or β subunits) and matriglycan synthesis on α-DG are frequently reduced in many different types of primary tumors (Table 1), these changes being associated with cancer progression and predictive of a poor outcome [85, 87, 88, 91, 103]. Additionally, and regarding β-DG, one of the most notable changes detected both in tumor samples and cancer cell lines is its fragmentation to yield polypeptides harboring different sizes, additional to that of the canonical β-DG of 43 kDa, of which proteoforms of ∼31 and 26 kDa are attributable to proteolysis, and those between 43 and 38 kDa to misglycosylation [109]. It must be taken here into account that the molecular mass of the β-DG core peptide is 26 kDa. Of such proteoforms, the β-DG fragment of ∼31 kDa was the most frequently found in cancer, including oral, colon, prostate, cervix, breast, glioma and leukemia primary tumors and/or cell lines [66, 67, 79, 85, 88, 90, 99, 100, 104, 106, 109, 110]. This proteoform is generated upon removal of the β-DG N-terminal extracellular domain by gelatinases, namely MMP-9 and - 2 [100, 101, 164], which renders β-DG unable to interact with α-DG and would presumably promote release of the latter to the extracellular medium with consequent disruption of cell-ECM interaction. Proteolysis of β-DG to yield this fragment is enhanced in cancer as well as in certain muscular dystrophies [79, 100, 101, 104]. Also to note is the finding that the ∼31 kDa β-DG fragment phosphorylated on tyrosine (by c-Src) is usually present in the nucleus of patient tumor cells and cultured cell lines [77, 99, 109]. A relationship between cell density in culture and the appearance of the above set of sub-43 kDa β-DG species resulting from post-translational modification has been reported in the LNCaP, PC3 and DU145 prostate cancer cell lines [109]. Additionally, the overexpression and localization of β-DG in the cytoplasm could be favored by the expression of AGR2 and its interaction with proteins of the ECM, where AGR2 has been reported to disrupt cell-cell adhesion and to promote the formation of invasive structures [165], as detected in the breast cancer cell lines MCF7 and MDA-MB-453 [110].

Accumulating evidence indicates that β-DG is a functionally-versatile protein normally exerting a number of functions from its location at the plasma membrane as part of the DGC, and also inside the nucleus. At the plasma membrane β-DG serves as a multifunctional scaffold able to bind, by virtue of its cytoplasmic domain, to various proteins involved in different signal transduction pathways [28, 53]. These include the adaptor protein Grb2, which has a role in cytoskeleton organization and is involved in malignant transformation [166], and other components of the Ras/Raf/MAPK cascade, including MEK and active ERK. These interactions take place at specialized structures at the cell surface known as membrane ruffles and focal adhesions (in the case of MEK and ERK, respectively), the latter including the actin-rich structures of migrating cells named filopodia [23, 51, 52, 140, 167]. Sequestering by β-DG of Grb2, MEK and ERK in response to extracellular cues, such as integrin engagement or α-DG-mediated cell adhesion to laminin, can hinder activation of the Ras/Raf/MAPK cascade, thereby modulating cell proliferation [51, 53, 85, 102]. Also, β-DG, upon becoming phosphorylated on tyrosine in response to cell adhesion to laminin, can recruit a number of SH2 domain-containing adaptor proteins, such as Shc, Nck and kinases of the Src family, this allowing β-DG to exert a key function in the modulation of various signaling cascades [28].

Given that β-DG is often found fragmented, and its expression reduced or undetected in many human carcinomas, these alterations can have different effects on the role exerted by β-DG on different signal transduction pathways, resulting in the growth-rate and metabolic changes observed in tumorigenic cells [19, 97, 100]. Such β-DG alterations can also provoke disruption of ECM-cytoskeleton interactions, this favoring cell migration and invasiveness [167]. This has been documented in human prostate tumor cells, where β-DG loses its expression and undergoes proteolysis along with tumor advance, this likely accounting for dysregulation of β-DG function leading to altered cell adhesion and motility and β-DG proper signaling, thereby contributing to the cancer phenotype [99, 109]. In this context, by exogenously overexpressing a *DAG1* cDNA a decrease of tumorigenicity was achieved in the prostate cancer LNCaP cell line without a recovery of α-DG matriglycan levels [88]. This strongly suggested that β-DG can control cell proliferation independently of α-DG, by virtue of its ability to bind, and sequester, signaling molecules as indicated.

In addition to its critical roles in cell adhesion, signaling and cytoskeletal remodeling played at the plasma membrane, β-DG is often found in the nucleus. To reach this compartment, β-DG undergoes endocytosis from the plasma membrane, in a process independent of α-DG ligand binding and that is positively modulated by its phosphorylation on Tyr-892, which disrupts its interaction with dystrophin [72], being then retrogradely targeted both to the endoplasmic reticulum and to the nucleus (Fig. 1) [6, 74]. Translocation through the nuclear pores is carried out upon recognition of its functional nuclear localization sequence (NLS; aas 776–782) by the importin system [6, 74, 168]. Once in the nucleus, β-DG forms specific protein assemblies at the nuclear envelope, more precisely at the inner nuclear membrane, and in the nuclear matrix, where it is forms part of a DGC-like nuclear complex bearing many components of the plasma-membrane DGC, including dystrophin [54, 169, 170]. Also, β-DG is found in a number of different nucleoplasmic compartments, including splicing speckles, Cajal bodies and nucleoli [54, 55, 169]. At these nuclear locations β-DG is thought to play multiple roles in the orchestration of nuclear architecture and activity, including nuclear envelope structure and function, and indirect modulation of gene expression. Furthermore, β-DG can also shuttle back to the cytoplasm by virtue of its nuclear export signal (NES; aas 763–772) located in its transmembrane domain, through an exportin-mediated process, being its nucleocytoplasmic trafficking tightly regulated [55]. All this complexity underlying β-DG multiple locations, multifunctionality and dynamics is thought to contribute to regulation of cell division and/or cell differentiation in response to events occurring at the plasma membrane, likely in a sophisticated fashion whose understanding still requires substantial investigation [54, 74].

Several instances of abnormal β-DG localization and/or function in the nucleus presumably associated with tumorigenesis are known, although the molecular mechanism underlying the potential role played by β-DG in this subcellular compartment, observed only under some physiopathological conditions, has not been clarified yet. Significant differences are found in the nuclear levels of β-DG in a number of breast cancer cell lines, independently of the status of glycosylation or ligand binding of the α-DG subunit [6]. In AML it has been reported that, despite the downregulation of the *DAG1* transcript, the immunolocalization pattern of α-DG and β-DG in the plasma membrane was very similar when comparing control (CD34^+^) cells from healthy individuals with primary blasts from AML patients or with the HL-60 and Kasumi-1 cell lines [77]. However, the presence of full-length (43 kDa) phosphorylated β-DG was observed only in the nucleus of tumor cells, in both AML patients and the two cell lines, and not in control cells from healthy individuals [77]. Furthermore, it was determined that α-DG was hypoglycosylated both in AML patients and the two cell lines (Tables 1, 2), which was associated with a downregulation of *POMT1*, *POMT2*, *POMGNT1*, *FKTN*, *FKRP* and *LARGE1* genes in the two cell lines (Table 4), and either upregulation or downregulation of these genes in cells of patients (Table 3), as mentioned above. It is thus tempting to speculate that the presence of phosphorylated β-DG in the nucleus of these cells could indirectly alter the expression of DGP-associated genes, with ensuing loss of α-DG matriglycan and increase of cell proliferation.

β-DG proteoforms of 43 kDa and smaller in size have been detected in both the cytoplasmic and nuclear fractions of healthy and prostate tumor tissue [99]. However, a higher translocation to the nucleus of the Tyr-phosphorylated β-DG ∼31 kDa species was solely found in LNCaP cells, in contrast to the control, non-tumor cell lines also studied. *In silico* analyses indicate that β-DG lacks DNA-binding motifs [52], and hence that it is not likely to act as a transcription factor itself, but instead might indirectly function by binding other, to be identified, regulatory proteins in the nucleus [26, 99] or act by modulating mRNA processing or transport [168]. In this light, three genes have been identified by microarray analysis whose mRNA levels became significantly upregulated upon nuclear targeting of a ∼31 kDa β-DG construct (in comparison to the same construct lacking an active NLS) in LNCaP cells. These genes encoded RGS20 (regulator of G-protein signaling 20), BAAT (bile acid-CoA:amino acid *N*-acyltransferase, with an unknown role in signal transduction) and ETV1 (ETS translocation variant 1) [99]. The latter, interestingly, is an androgen-inducible activating transcription factor with an established role in the regulation of prostate growth. In this light, it has been found that disruption of *ETV1* expression (by siRNA transfection) strongly compromises the invasive capacity of both androgen-dependent (LNCaP) and -independent (C81) cells, suggesting that ETV1 plays an important role in prostate cancer metastasis [171]. Furthermore, oncogenic *ETV1*, which originates by chromosomal rearrangement, is capable of activating the Ras/Raf/MAPK pathway in the absence of activated, phosphorylated ERK [172]. Therefore, the combined effects of (i) α-DG hypoglycosylation and loss of laminin binding; (ii) β-DG fragmentation carried out, in part, by MMPs with consequent changes in the scaffolding of MAPK signaling; and (iii) nuclear translocation of β-DG resulting in upregulated *ETV1* transcription and, in turn, of MAPK-dependent signaling leading to an increase in cell migration, could represent a positive feed-back mechanism driving prostate cancer progression [99]. Since ETV1 acts as an oncoprotein in a wide variety of human cancers, including Ewing sarcoma, colorectal, breast, melanoma and others [173], this mechanism might well be extrapolatable to other tumor types.

Finally, the proposal has been raised by our group [174, 175] and others [138, 142, 176] that DGP-associated proteins, such as POMGNT1, fukutin and FKRP, could act (abnormally) inside the nucleus of tumor cells to glycosylate yet-to-identify proteins with a role in cell proliferation, adhesion and/or migration, including intranuclear signaling proteins and transcription factors, thereby modulating their function. Further research is needed to clarify this and many other aspects of the involvement of DG and DGP-associated proteins in cancer pathogenesis.

## Concluding remarks

A deficient or undetectable glycosylation of α-DG is consistently found in a wide variety of human tumor types and cancer cell lines. Also, a correlation exists with very few exceptions between hypoglycosylation of α-DG and loss of laminin binding, and thereby of cell anchoring to the ECM of the tissue in which the tumor originates. Although this is not clearly a causative or initial event during the origin of the tumor, it is a significant factor contributing to tumorigenesis and the malignant phenotype. Whether cause or consequence, it leads to an increase in cell growth and proliferation, together with the acquisition of migration and invasion properties, eventually resulting in metastasis and malignancy. Additionally, β-DG anomalous downregulation, fragmentation and/or translocation to the nucleus is increasingly clear to contribute to the tumorigenic process.

In a limited number of studies low levels of glycosylated α-DG (i.e., of matriglycan in particular) have been demonstrated to parallel an underexpression of *DAG1* mRNA and/or α-DG (core) protein. However, in an ample set of human tumors and cell lines α-DG hypoglycosylation has been linked to downregulation of genes associated with DGPs or their encoded proteins, which are enzymes participating in the complex process of α-DG *O*-mannosyl glycosylation. Indeed, knockdown of some of these genes, including *POMT1, B4GAT1* and *LARGE1*, or of the DG-encoding gene itself, *DAG1*, results in a loss of laminin-binding glycans with a concomitant increase in cell migration involving the activation of AKT and ERK. Conversely, forced overexpression of *DAG1* rescues laminin-binding ability in a number of cell lines, with inhibition of cell-cycle progression, loss of anchorage-independent growth and decrease of tumorigenicity. Downregulation of DGP-associated genes in tumors and cell lines has been attributed to their silencing through promoter methylation or to transcriptional repression, as it has been demonstrated for *LARGE1* and *LARGE2* genes, respectively. It is thus concluded that hypoglycosylation of α-DG is an event associated with tumor origin and/or progression, and likely necessary for the detachment of cancer cells from their ECM.

A relationship has also been found between α-DG hypoglycosylation and loss of both E-cadherin expression and *O*-mannosyl glycosylation, in favor of aberrant *N-*glycosylation mediated by GnT-Va. The silencing of the E-cadherin encoding gene, *CDH1*, is mediated by the EMT-promoting transcription factors ZEB1 and Snail, two regulators that are overexpressed in many cancers, and which act jointly to repress both *CDH1* and *LARGE2* mRNAs. This translates into reduced glycosylated α-DG and E-cadherin levels, with consequent loss of cell-cell contacts, a feature of abnormal, adult EMT that has been associated with invasiveness and tumor metastasis. In this context, it is known that DG is lost together with E-cadherin during metastatic spread, and that both molecules are reexpressed during a process resembling MET when colonizing a secondary target organ.

There are some types of tumors such as kidney, liver, breast, and AML in which several DGP-associated genes are usually found to be altered at the same time (Tables 3 and 4). We could collectively define this kind of tumors as the ‘dystroglycan axis’, whose therapeutic implications would be interesting to study in the near future. Furthemore, in solid tumors and derived cell lines (i.e., leaving aside leukemia) genes directly involved in the synthesis of core M3 glycan and matriglycan, such as *POMGNT2*, *CRPPA*, *B4GAT1*, *LARGE1* and *LARGE2* and/or their encoded proteins, are found downregulated (Fig. 2). Given that this structure is crucial for α-DG function, and hence for cell attachment to the ECM, these genes play a likely role as tumor suppressors. On the other hand, genes acting at earlier steps in the α-DG *O-*mannosyl glycosylation pathway, including *DOLK*, *DPM1*/*2*/*3*, *POMGNT1*, *B3GALNT2*, *POMK* and *FKTN*, are generally found upregulated in solid tumors at the mRNA and/or protein levels (Fig. 2), and therefore exhibit a pro-oncogenic behavior. Their tumorigenic action appears to be exerted through modulation of signaling pathways (PI3K/AKT/GSK-3β, ERK/MAPK) or gene expression (integrins, β-catenin, *MYC*, etc.), by molecular mechanisms that are beginning to emerge, as discussed above, and that are in need of further research.

While α-DG interaction with laminin is important for cellular polarization and maturation, β-DG itself plays a function as a signaling molecule and indirect modulator of gene expression independently of α-DG glycosylation, aimed at regulating cell proliferation, which must be taken into account in the field of cancer pathogenesis. As part of the DGC, β-DG acts as a scaffold or multifunctional adaptor in the cytoplasm, binding to and sequestering Grb2 and other signaling proteins of the Ras/Raf/MAPK pathway, and eventually bringing about inhibition of cell division-stimulatory signals and/or enhancement of growth-inhibitory signals, thereby playing a role itself as a tumor suppressor. However, the β-DG tumorigenic potential becomes evident when it is found underexpressed or fragmented, by MMPs and other unidentified proteases, into lower-molecular-weight proteoforms, of which the ∼31 and (the full-length) 43 kDa species are found phosphorylated in the nucleus. Interference of normal cytoplasmic signal transduction by these β-DG species results in dysregulation of cell proliferation and differentiation by not well known mechanisms. Also, β-DG forms assemblies at the nuclear envelope and matrix, acting as a scaffolding protein involved in the organization of nuclear architecture and the modulation of transcriptionally-active regions as a resident component of different intranuclear compartments. It is thus tempting to suggest that, when dysfunctional, β-DG could act to downregulate DGP-associated genes in some human cancers, this resulting in decreased α-DG matriglycan levels and ensuing enhanced cell proliferation. The transcription factors with whom β-DG interacts physically or functionally to modulate the cell division cycle or cell differentiation remain essentially unknown, with the exception of ETV1, a transcriptional regulator with an involvement in prostate cancer metastasis.

**Fig. 3:**
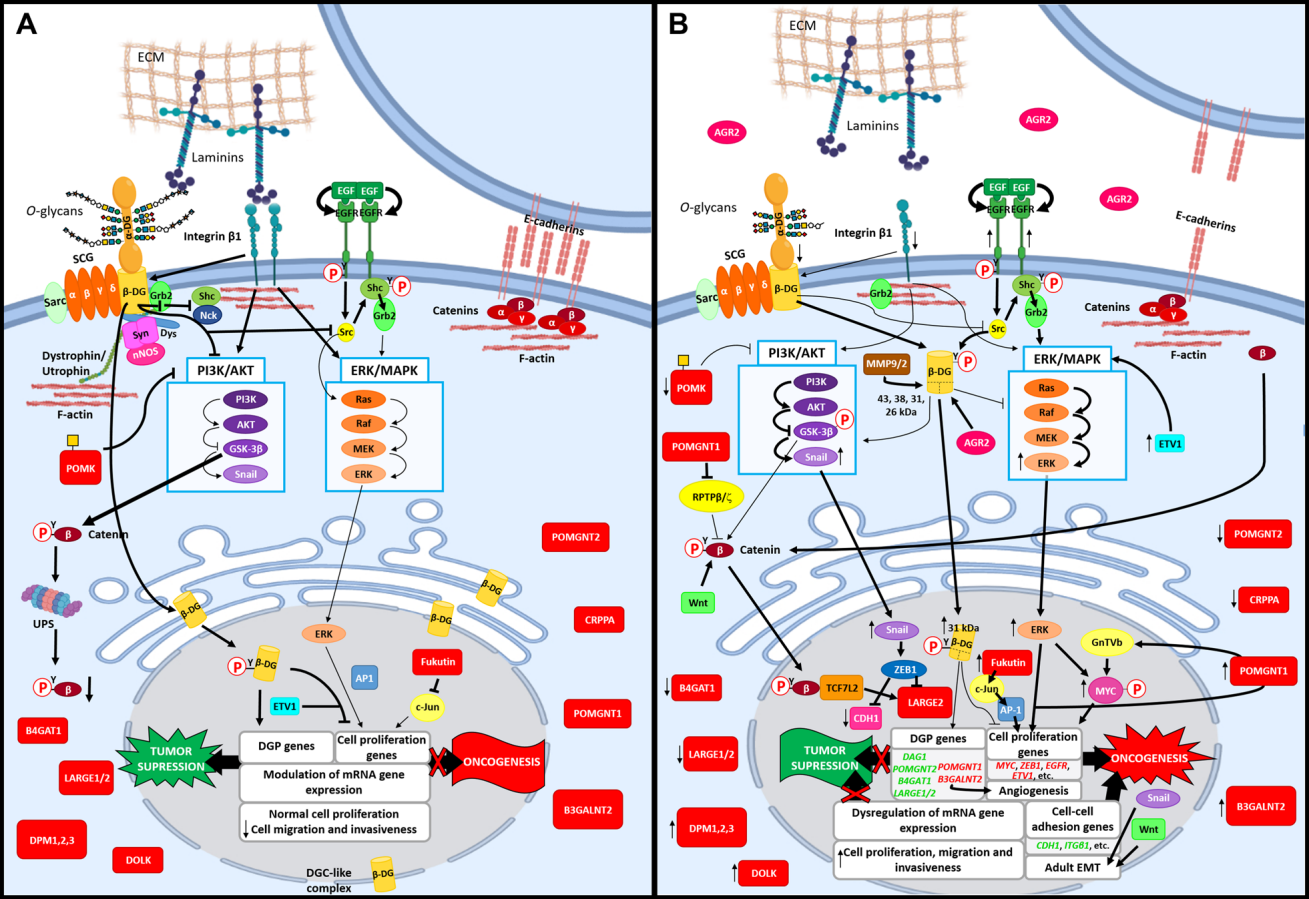
Alterations in dystroglycan-related signal transduction and gene expression in tumor cells. The two subunits of dystroglycan, α-DG and β-DG, are constituent polypeptides of the DGC located at the plasma membrane. In normal cells (**A**) α-DG glycosylation carried out by DGP-associated proteins is crucial for the anchorage of cells to the ECM, and both integrins and cadherins provide cell-cell adhesion, whereas β-DG contacts the actin cytoskeleton via dystrophin and also acts as a scaffold/multifunctional adaptor in the cytoplasm. Upon cell-ECM interaction and integrin β1 engagement β-DG becomes Tyr-phosphorylated, thereby inhibiting components of the ERK/MAPK and PI3K/AKT pathways through its binding to, and sequestering of, signaling proteins such as Grb2, Src and others. In tumor cells (**B**) α-DG hypoglycosylation, sometimes found associated with the underexpression of particular DGP-associated proteins or α-DG core protein itself, prevents α-DG binding to laminin with consequent loss of cell attachment to the ECM, whereas E-cadherin downregulation hinders cell-cell adhesion. Also, underexpressed β-DG in tumor cells is unable to prevent activation of ERK/MAPK and PI3K/AKT pathways. Signaling through the ERK/MAPK cascade leads to translocation into the nucleus of MAPKs (such as ERK) and consequent activation of genes promoting cell division and oncogenesis (such as the *EGFR*, *MYC* and *ZEB1*). Also, activation of the PI3K/AKT pathway leads to inhibition of GSK-3β following its phosphorylation by AKT and subsequent activation of Snail, this entering the nucleus and downregulating (through ZEB1) E-cadherin, integrin β1 and LARGE2-encoding genes and promoting adult EMT. In normal cells (**A**) β-DG also undergoes cleavage by MMPs and phosphorylation by Src, this allowing its dissociation from α-DG and dystrophin and its retrograde targeting to the endoplasmic reticulum and the nucleus, where it localizes both at the nuclear envelope (forming part of a DGC-like complex) and at defined nuclear bodies, where it modulates nuclear structure and activity, including the expression of genes involved in normal cell proliferation. Low levels of β-DG and its abnormal fragmentation in tumor cells (**B**) prevent its normal modulation of DGP-associated and other genes with a tumor-suppressor role mediated by ETV1 and other transcription factors. However, a subset of other DGP-associated proteins become instead upregulated in tumor cells (**B**), among which the best known, POMGNT1, inhibits the RPTPβ/ζ phosphatase through its glycosylation carried out by GnT-Vb in the cytoplasm, upregulates *MYC* and further promotes EMT through the *N*-glycosylation of N-cadherins. The absence of β-catenin inhibition by GSK-3β and RPTPβ/ζ promotes its tyrosine phosphorylation and migration to the nucleus, where (tyrosine-phosphorylated) β-catenin contributes to upregulate LARGE2 together with Wnt-target, EMT-promoting genes. The loss in tumor cells of cell-ECM and cell-cell adhesion together with EMT occurrence and abnormal function of β-DG in the nucleus, likely resulting in dysregulation of genes involved in normal cell division, favors abnormal cell proliferation, migration and invasiveness, and this paving the way to tumorigenesis. Created with BioRender.com

To conclude, the panorama of α-DG glycosylation and β-DG role as preventers of cell detachment from the ECM, uncontrolled cell division and invasiveness, as shown in Fig. 3, provides a framework in which new molecular biomarkers of cancer diagnosis and prognosis can be identified, and in which new therapeutic targets can be sought, these constituting the bases for new, more specialized treatments. A good example of DGP-associated protein combining both potentials is POMGNT1, whose increased levels correlate well with malignancy, tumor grade, resistance to chemotherapy and low patient survival in human gliomas, and whose intervention, either genetic or pharmacological, could be a good strategy targeting defined cancer types. Conversely, underexpressed *LARGE1* and *LARGE2* could be potential targets for therapies enhancing their levels. Also, strategies should be investigated aimed at increasing DG expression, and hence α-DG glycosylation function in cell-cell and cell-ECM adhesion and β-DG tumor-suppressor function in a joint fashion. The unraveling of all interacting partners of α-DG and β-DG at their different locations in and outside the cell should eventually constitute a source of new targets of therapies against human cancers. Finally, virtually all the genes and proteins dealt with in this review should be included in NGS- or microarray-based transcriptomic panels or protein microarrays aimed at detecting alterations in their expression in human tumor samples with diagnostic and/or prognostic value.

## Data Availability

Not applicable.
